# A Comprehensive Survey on Security and Privacy for Electronic Health Data

**DOI:** 10.3390/ijerph18189668

**Published:** 2021-09-14

**Authors:** Se-Ra Oh, Young-Duk Seo, Euijong Lee, Young-Gab Kim

**Affiliations:** 1 Miro Corporation, Incheon 21988, Korea; sroh@gomiro.com; 2 Department of Computer Engineering, Inha University, Incheon 22212, Korea; mysid88@inha.ac.kr; 3 Department of Computer Science, Chungbuk National University, Cheongju 28644, Korea; kongjjagae@cbnu.ac.kr; 4 Department of Computer and Information Security, and Convergence Engineering for Intelligent Drone, Sejong University, Seoul 05006, Korea

**Keywords:** security concerns, security requirements, security solutions, e-health data, medical devices, medical networks, edge computing, fog computing, cloud computing

## Abstract

Recently, the integration of state-of-the-art technologies, such as modern sensors, networks, and cloud computing, has revolutionized the conventional healthcare system. However, security concerns have increasingly been emerging due to the integration of technologies. Therefore, the security and privacy issues associated with e-health data must be properly explored. In this paper, to investigate the security and privacy of e-health systems, we identified major components of the modern e-health systems (i.e., e-health data, medical devices, medical networks and edge/fog/cloud). Then, we reviewed recent security and privacy studies that focus on each component of the e-health systems. Based on the review, we obtained research taxonomy, security concerns, requirements, solutions, research trends, and open challenges for the components with strengths and weaknesses of the analyzed studies. In particular, edge and fog computing studies for e-health security and privacy were reviewed since the studies had mostly not been analyzed in other survey papers.

## 1. Introduction

The advancement of modern technologies, such as sensors and cloud computing, has completely changed conventional healthcare systems. Such systems can demonstrate the strong potential of next-generation healthcare services after digitizing paper-based medical records. Individuals’ health conditions can be remotely sensed by medical devices, transmitted by medical networks, and processed by the edge, fog, and cloud computing. Innovative healthcare systems that can improve quality of life will become more essential for various smart healthcare services such as remote monitoring, diagnosis, treatment, and prescription based on personal electronic health (e-health) data. However, the modern e-healthcare system is a double-edged sword. While it gives us advanced healthcare services, security concerns have increasingly emerged.

E-health data are some of the most private information for individuals. Regulations for privacy protection such as the Health Insurance Portability and Accountability Act (HIPPA) [1] and General Data Protection Regulation (GDPR) [2] have been established to enhance the governance of healthcare data; however, e-health data has been frequently breached. In addition, as the accessibility and usability of e-health data increase, its security attack vectors have also been widening. Over the last decade, 1.5 million medical devices have been compromised due to software vulnerabilities and their wireless connection [3], and cloud computing services that store and process e-health data have become a target for big e-health data. According to the Protenus Breach Barometer, 41.4 million patients’ records were breached in 2019 [4].

Therefore, security and privacy issues must be explored to prevent breaches of e-health data. In particular, security concerns, requirements, and solutions must be identified to properly study how to secure e-healthcare systems. Consequently, the primary goal of this paper is to survey security and privacy studies to identify security concerns, requirements, and solutions. Specifically, because modern e-healthcare systems generally consist of several components (i.e., e-health data, medical devices, medical networks, and edge, fog, and cloud computing) that have their own characteristics, security concerns, requirements, and solutions are surveyed by component. In addition, this paper presents recent research trends and open challenges for each component.

During the last five years, many survey papers focusing on the security and privacy of e-health data have been published; however, there has been no comprehensive survey of an overall e-healthcare system, such as e-health data, medical devices, medical networks, and edge/fog/cloud computing that senses, transmits, stores, and processes e-health data. There have been some surveys focusing on specific components of e-healthcare systems, that is, e-health data security [5,6,7,8], medical device security [3,9,10,11,12], and medical network security [13,14]. Other studies [15,16,17,18] have aimed at more than one component of the e-healthcare system. However, the security and privacy issues for all components have not yet been surveyed. To the best of our knowledge, this is the first comprehensive survey paper to identify security concerns, requirements, solutions, research trends, and open challenges for each component of the e-health system consisting of e-health data, medical devices, medical networks, and edge, fog, and cloud computing. The main contributions of this paper are as follows:

- A comprehensive survey on the security and privacy issues for e-health data, medical devices, medical networks, edge, fog, and cloud computing;
- Identification and taxonomies of the security concerns, security requirements, and security solutions for e-health data, medical devices, medical networks, and edge/fog/cloud computing;
- Analysis and identification of the strengths and weaknesses of the surveyed studies;
- Identification of the research trends and open challenges for each component (i.e., e-health data, medical devices, medical networks, edge, fog, and cloud computing) of e-health systems.

In Section 2, the background of this paper is described in terms of research questions, search strategy, target domains, and related works. Section 3 then provides security concerns, requirements, and solutions by reviewing recent security and privacy studies for e-health data. Similarly, for the medical device, medical network, and edge/fog/cloud computing, Section 4, Section 5 and Section 6, respectively, discuss security concerns, requirements, and solutions. Then, Section 7 discusses research trends and open challenges for the components of modern e-health systems. Finally, Section 8 concludes this survey.

## 2. Background

This section presents a method of searching and selecting security and privacy studies related to e-health data. Then, four main components (i.e., e-health data, medical devices, medical networks, and edge/fog/cloud) of modern e-health systems are identified as the target domains of this survey. Finally, related works, which are existing security and privacy surveys in the medical domains, are also analyzed.

### 2.1. Method

**Figure 1:**
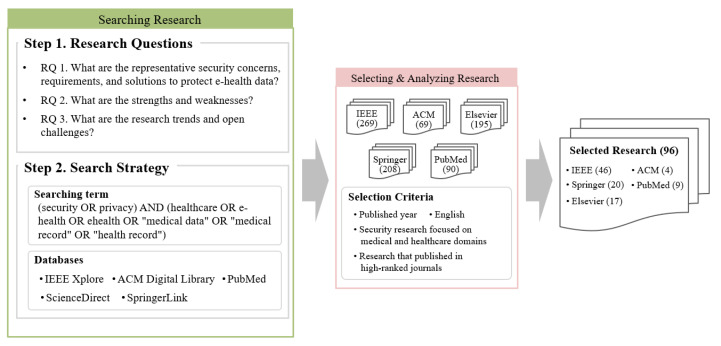
Overview of the literature review procedure.

In this paper, we created and followed a method based on the systematic literature review (SLR) approach [19] to search and select studies that focus on security and privacy issues related to e-health data. Figure 1 denotes the literature review procedure.

The primary goal of this survey is to highlight the security concerns, requirements, and solutions, research trends, and open challenges for e-health data. For a consistent and meaningful survey, we carefully formed the following key research questions (RQs). Note that the target domains of this paper are described in Section 2.2 based on the analysis of the selected studies.

- RQ 1: What are the representative security concerns, requirements, and solutions to protect e-health data for each target domain?
- RQ 2: What are the strengths and weaknesses of the surveyed studies for each target domain?
- RQ 3: What are the research trends and open challenges for each target domain?

To answer the questions, we selected general search keywords such as “security”, “privacy”, and “healthcare” as described in Figure 1 for a comprehensive survey. We compiled 831 studies from the international literature databases (i.e., IEEE Xplore, ACM Digital Library, ScienceDirect, SpringerLink, and PubMed). Then, following selection criteria were considered to select key studies for solving our questions.

- SC 1: Studies must have been published within five years;
- SC 2: Studies must use English;
- SC 3: Studies must focus on medical or healthcare domains. There were various security and privacy studies in diverse environments such as Internet of Things (IoT), edge, fog, and cloud; however, we excluded studies that did not focus on the medical or healthcare domains;
- SC 4: Studies must focus on technical research. we excluded some studies regarding medical policies, social sciences, etc.;
- SC 5: Journals should be ranked top 15% in Journal Citation Reports (JCR). If journals were not ranked in the JCR, it should have around 0.8 or higher SCImago Journal Rank (SJR). However, medical journals were selected even if they were ranked around the top 50% in JCR or had 0.4 or higher SJR because of their expertise.

In case of similar works, we compared their published date, originality, and overall quality. After selection, we finally obtained 96 studies that focus on security and privacy for e-health domains. Table 1 shows which journals published the surveyed studies; the impact factors (IFs) and SJRs in Table 1 correspond to 2020.

### 2.2. Target Domain

**Figure 2:**
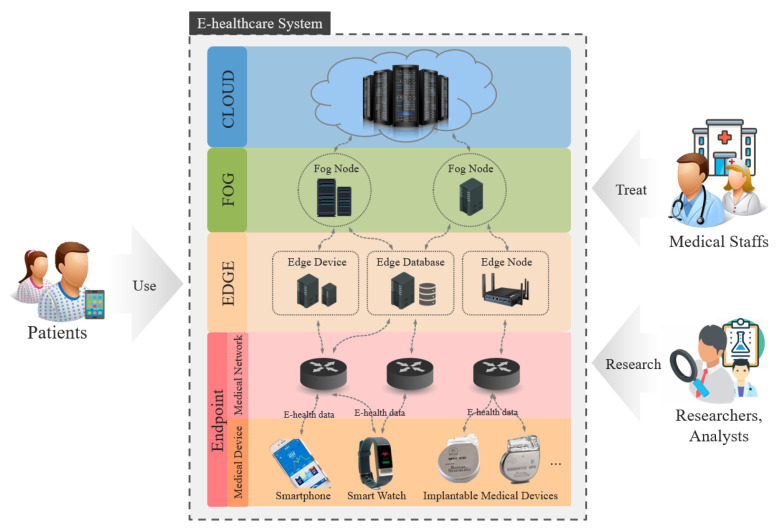
Overview of e-health system and the target domains.

Driven by diverse technical advancements, studies on the security and privacy of e-health data have been conducted with different target domains such as medical devices and networks. Therefore, to comprehensively survey the security and privacy issues of protecting data with the consideration of overall domains, we analyzed existing studies to identify the common components of modern e-health systems as the target domains of this survey. Then, we surveyed the studies according to the domains to identify security concerns, requirements, solutions, research trends, and open challenges for each domain. Figure 2 shows an overview of the e-health system and the target domains of this survey.

In a modern e-health system, patients’ e-health data can be generated by medical devices, transmitted via medical networks, stored, and processed in edge/fog/cloud. Therefore, to comprehensively cover the security and privacy of e-health data, e-health data and the surrounding environments (i.e., medical devices, medical networks, and edge/fog/cloud computing) are the main target domains of this survey.

### 2.3. Related Work

Based on the searching and selection method described in Section 2.1, we found 15 security and privacy survey papers in medical/healthcare domains. Table 2 shows the papers and their target domains.

Most surveys focused on one or two specific domains. Some of the surveys [5,6,7,8] studied the security and privacy of e-health data such as electronic health record (EHR) and genomic data, and some surveys [3,9,10,11,12] focused on the security and privacy of medical devices. In addition, two surveys [13,14] focused on the security and privacy of medical networks such as the Internet of Medical Things (IoMT) and Body Area Network (BAN), and there were security and privacy surveys on e-health challenges in the cloud, mobile healthcare (mHealth) systems, electronic health services, and the medical domain [15,16,17,18]. Here follows a brief summary of each survey.

Several studies investigated security and privacy factors for e-health data. Kruse et al. [5] collected 25 journals from PubMed, CINAHL, and ProQuest Nursing and Allied Health Source, and analyzed the journals to investigate security techniques for EHRs. The security techniques were analyzed and categorized into three themes: administrative safeguard (e.g., risk management and system security evaluation), physical safeguard (e.g., physical access control and workstation security), and technical safeguard (e.g., authentication, access control, audit, data encryption, and firewall). Abouelmehdi et al. [6] surveyed security and privacy challenges for big healthcare data. To accomplish the survey, several studies including security factors (i.e., authentication, data encryption, data masking, access control, de-identification, and identity-based anonymization) were analyzed. In addition, Mohammed et al. [7] and Aziz et al. [8] surveyed security and privacy for genomic data. Mohammed et al. identified three types of attacks (i.e., identity tracing, attribute disclosure, and completion attacks) to genome privacy. They also classified genome privacy-preserving solutions (e.g., differential privacy and homomorphic encryption) that is related to the attacks. Aziz et al. [8] discussed privacy problems on genome data and reviewed privacy-preserving solutions regarding homomorphic encryption, Garbled circuit, secure hardware, and differential privacy.

There are survey papers related to medical devices. Zheng et al. [9] surveyed challenges for securing wireless implantable medical devices (IMDs). In the paper, they discussed security requirements, security solutions supporting emergency access, and lightweight security schemes for access control. Wu et al. [10] specifically surveyed access control schemes for IMDs. They reviewed the existing studies for IMD access control and classified the IMD access control schemes into four groups (i.e., direct access control with preloaded keys, direct access control with temporary keys, indirect access control via a proxy, and anomaly detection-based schemes). Yaqoob et al. [3] surveyed studies for medical devices, but they focused on security vulnerabilities, attacks, and countermeasures of the networked medical devices. In the study, a network model and attack vector are described, then security vulnerabilities, attacks, and countermeasures were analyzed for the medical device products. In addition, Kintzlinger et al. [11] analyzed the security of personal medical devices (PMDs) and their ecosystems. They provided a specific attack flows in the PMDs and its ecosystem. They also surveyed possible attacks and mechanisms to protect the attacks. AlTawy et al. [12] also surveyed security attacks and threats of medical devices, but they focused on various types of security tradeoffs between security, safety, and availability.

Yaacoub et al. [13] and Sun et al. [14] surveyed security and privacy for IoMT. Yaacoub et al. [13] presented the components of IoMT (e.g., the types of IoMT, devices, and protocols), and analyzed the security issues, concerns, challenges, attacks, and countermeasures in the IoMT. Sun et al. [14] also surveyed security and privacy-related studies for IoMT. They identified 14 security and privacy requirements for the IoMT on several levels: data level, sensor level, personal server level, and medical server level.

Moreover, several studies were investigated the security and privacy of e-health challenges in the cloud environments, mobile healthcare system, electronic health services, and medical domain [15,16,17,18]. Chenthara et al. [15] reviewed security and privacy challenges and approaches of e-health solutions for electronic health records (EHR) in the cloud environment. In particular, they identified security and privacy requirements for e-health data and analyzed various studies focused on privacy-preserving approaches using cryptographic techniques (i.e., symmetric key encryption, public key encryption, and a few alternative cryptographic primitives) and non-cryptographic techniques (i.e., access control). In addition, Yüksel et al. [16] conducted a survey on the security and privacy for electronic health services (EHSs). They particularly categorized recent studies into six groups (i.e., architecture, access control, emergency, sharing, search, and anonymity), and presented analyzed results and open challenges based on the research groups. Wazid et al. [17] surveyed security protocols for mHealth. They discussed security requirements, issues, and threats for mHealth systems, and presented a taxonomy of security protocols for mHealth. They also performed a comparison of the protocols in terms of computation cost and communication cost. Razaque et al. [18] introduced a survey on security vulnerabilities and attacks for the medical domain. The security vulnerabilities and attacks were analyzed according to the dataflow (i.e., patient registration, data collection, storing and utilizing the data) in the medical domain.

We found more than 40 survey papers. However, only 15 surveys are analyzed in this paper because the others are not related to medical domains or not focused on security and privacy issues. Moreover, according to Table 2, no surveys considered the overall components of modern e-health systems (i.e., e-health data, medical device, medical network, and edge/fog/cloud computing). Therefore, this survey focuses on the four major components of e-health systems to comprehensively identify the security concerns, requirements, solutions, research trends, and open challenges for each.

## 3. E-Health Data

This section presents security concerns, requirements, solutions, research trends, and open challenges for the security and privacy of e-health data.

### 3.1. Overview

**Figure 3:**
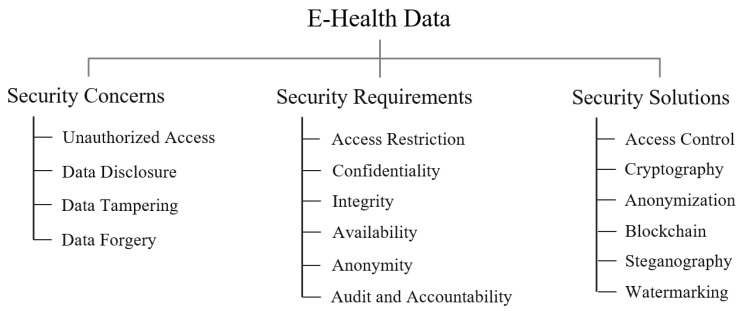
A taxonomy on the security and privacy for e-health data.

The initial goal of this section was to explore various security and privacy studies on e-health data; however, there were no sufficient security or privacy studies focusing on e-health data itself. Most studies focused on proposing new security solutions such as cryptography and authentication required to protect the e-health data. Therefore, the contents of this section were collected and analyzed based on a few studies for e-health data and the various studies in diverse medical/healthcare domains such as medical devices and networks that mention e-health data security and privacy. Figure 3 shows a taxonomy for security concerns, requirements, and solutions for e-health data.

### 3.2. Security Concern, Requirement, and Solution

According to our survey, most studies focused on other target domains such as medical network and cloud computing rather than e-health data itself. Therefore, we collected and analyzed the security concerns, requirements, and solutions for e-health data from diverse studies in different domains that partially mentioned the security and privacy issues of e-health data. A few dedicated security and privacy studies on e-health data are also analyzed in this section.

#### 3.2.1. Security Concern

E-health data are some of the most critical and private information in modern society. However, security concerns for the data have emerged because of insufficient security. For example, attackers can exploit some security vulnerabilities of e-health systems to breach the data and forge their identities to deceive the systems. Tampering e-health data becomes a critical issue since it can pose medical accidents. Four security concerns on e-health data, that were commonly mentioned in the surveyed studies are as follows.

**Unauthorized access.** Various security vulnerabilities in medical devices, networks, and platforms, such as edge, fog and cloud, are at risk of unauthorized access. By using their vulnerabilities, an attacker can access the system to capture sensitive e-health data.

**Data disclosure.** Data disclosure can occur throughout the e-healthcare system such as medical devices, networks, and edge/fog/cloud platforms because of their security vulnerabilities or an administrative mistake. E-health data are an attractive target for attackers since it is very valuable. According to AlTawy et al. [12], a personal health record (PHR) on the black market was priced at around $50 USD, while a social security number was priced at around $3 USD.

**Data tampering.** Data tampering denotes the modification of data without appropriate authentication and authorization. This attack, which is also known as data modification, could be a critical security concern because tampered e-health data may have strong implications for patients.

**Data forgery.** E-health data or user identities can be forged to deceive legitimate service providers or impersonate others. By forging data, an attacker can compromise e-health systems, or a user with a malicious purpose can take inappropriate profits.

#### 3.2.2. Security Requirement

This section presents the six representative security requirements for e-health data. To securely protect e-health data with privacy preservation, security solutions for the data should consider proper security requirements. Data confidentiality, integrity, and availability are basic security requirements, and data anonymity is required for the patient’s privacy where the data are shared with someone who is not the data owner. Detailed descriptions of the security requirements are as follows.

**Access restriction.** Access restriction denotes the limitation of unauthorized access to assets such as e-health data, medical devices, and e-healthcare systems. This attack can be posed across entire medical domains; therefore, proper authentication and access control for each domain must be provided to prevent e-health data leaking.

**Data confidentiality.** In medical domains, the data confidentiality of e-health data is the most critical security requirement. An attacker can infringe data confidentiality by gathering data from various sources such as databases and networks. In particular, data can easily be captured from wireless medical networks such as IoMTs and wireless body area networks (WBANs). Data confidentiality is important; however, it can be breached when special cases happen in relation to critical patients.

**Data integrity.** Data integrity ensures that transmitted data are untampered with. This requirement is vital because doctors treat patients and prescribe medicine using the received data. The data integrity violation can directly influence patients’ health conditions. Therefore, receivers must verify whether transmitted data are untampered. Recently, Amato et al. [20] proposed a methodology for the validation of security and privacy policies in e-health systems.

**Data availability.** Databases and medical devices that store e-health data must be able to provide data regardless of time or location. Based on data availability, patients should be able to check their e-health data and medical staff should be able to use this data to treat their patients.

**Data anonymity.** Data anonymity should be provided by anonymizing e-health data to provide patient privacy when it has to be shared. In particular, there is a need to anonymize e-health data that is unrelated to a specific purpose and identity information about patients and medical staff that can be used to link anonymized data to identities.

**Auditability and accountability.** All information regarding e-health data such as generation time, owner, access records, and usage history must be recorded. By using recorded data (i.e., an audit trail), accountability can be satisfied to identify the person in charge when security incidents occur. These features, auditability and accountability, become critical if security attacks have implications for patients’ health conditions.

#### 3.2.3. Security Solution

Six security solutions (i.e., access control, cryptography, anonymization, blockchain, steganography, and watermarking) for the security and privacy of e-health data are presented in this section. E-health system must adopt access control systems to restrict unauthorized access, and the data stored in the system must be encrypted or anonymized using cryptography and data anonymization techniques. In addition, steganography and watermarking have widely been used to achieve medical image security, and blockchain has also been studied recently to ensure the integrity of the e-health data.

**Access control.** Access control is an indispensable security solution to protect e-health data by restricting unauthorized access. Therefore, many studies focused on the security and privacy of e-health data based on access control.

Dankar et al. [21] proposed a risk-aware secure framework that controls access to medical data using contextual information related to data requests. In the framework, to store e-health data, a risk evaluation module identifies the risk of the data, and an access control module determines the proper data protection level based on the risk. After selecting the protection level, a protection level application module re-identifies the data to store the data. The constraints to access the data are also decided using the data protection level if the data are requested.

In addition, there are studies to design access control frameworks using blockchains for secure management in e-health data [22,23,24]. However, the frameworks ensure several security benefits such as data confidentiality, integrity, availability, and accountability. Rajput et al. [22] applied an emergency scenario into an access control framework by defining some access rules for the emergent scenario. In the scenario, conditional permissions are used for the authorization of emergent medical staff. Shahnaz et al. [23] proposed a role-based access control framework to protect EHRs and focused on solving the scalability problem of blockchain based on the off-chain scaling method. Xu et al. [24] proposed Healthchain that controls access from medical staff by sharing symmetric keys between a user and the staff.

Furthermore, Section 4, Section 5 and Section 6 present other access control studies that focus on different target domains, that is, medical devices, networks, and edge/fog/cloud computing.

**Cryptography.** Cryptography has been widely used as an essential security solution to ensure several security requirements such as data confidentiality and integrity. Most studies adopted existing cryptography for basic purposes such as encryption and digital signatures, and only a few studies have proposed new cryptographic primitives, protocols, cryptosystems, and so forth. In general, well-known cryptosystems such as advanced encryption standard (AES) and Rivest–Shamir–Adleman (RSA) were utilized to protect e-health data in the studies, considering the different security requirements of the specific target domain. AES, developed by the National Institute of Standards and Technology (NIST) in 2001, is the most frequently used symmetric encryption technique [25]. Symmetric key techniques including AES are used in the medical/healthcare security research areas due to their fast encryption/decryption speed. On the other hand, RSA, developed in 1978 [26], is a public-key cryptosystem (PKC) that has two types of cryptographic key: a public key for encryption and a private key for decryption. RSA has been adopted for digital signatures rather than data encryption and decryption because it is neither a fast nor efficient cryptosystem.

Some studies focused on the security of digital image and communication on medicine (DICOM) [27,28,29]. Elhoseny et al. [27] simply applied AES and RSA to secure DICOM. Dzwonkowski et al. [28] and Parvees et al. [29] employed quaternion rotation and enhanced chaotic economic map (ECEM). They verified that the quaternion- and ECEM-based encryption schemes were more secure and efficient for medical image encryption than traditional cryptosystems such as AES.

In addition, Section 4, Section 5 and Section 6 contain more studies based on diverse cryptography schemes such as elliptic curve cryptosystem (ECC), attribute-based encryption (ABE), and certificateless public-key cryptosystem (CL-PKC) that considered security concerns and requirements for medical devices, networks, and edge/fog/cloud computing.

**Anonymization.** Data anonymization is a process that eliminates, generalizes, or replaces identifiable information from personal information [30]. For data anonymization, four traditional models (i.e., k-anonymity, l-diversity, t-closeness, and differential privacy) have been widely adopted in medical research areas.

K-anonymity is an anonymization model proposed by Sweeney in 2002 [31] that reduces the possibility of specifying sensitive attributes by producing k or more records composed of the same quasi-identifier. Therefore, if k-anonymity is satisfied, the probability of identifiability for a specific person will be <1/k. The higher the k value, the better the data anonymity. However, if the k value is too high, data usability decreases because it becomes more difficult to explore the correlation between anonymized data. An optimal k value should be found to provide an appropriate trade-off between data anonymization and usability.

Although the individual is not identified when k-anonymity is achieved, the more the sensitive attributes remain the same, the more likely they are to be re-identified. L-diversity therefore proposed by Machanavajjhala et al. in 2007 [32] can be adopted to prevent the limitation of k-anonymity by diversifying sensitive attributes. This reduces the possibility of re-identifying a specific individual with one or more sensitive attributes. As with k-anonymity, the higher the l value, the better the data anonymity.

If there is bias or patterns in anonymized data, personal privacy may be exposed by means of these biases and patterns. In other words, even if k-anonymity and l-diversity are satisfied, privacy can be disclosed using the distribution of sensitive attributes. Therefore, T-closeness proposed by Li et al. in 2007 [33] measures data distributions to prevent data from being closed in a specific part.

K-anonymity and l-diversity have the limitation that sensitive attributes can be exposed if an attacker has experience exploiting vulnerabilities. Therefore, differential privacy was proposed by Dwork in 2006 [34] to mitigate the limitations of k-anonymity and l-diversity. This mathematical model prevents an attacker from inferring a specific individual with statistical data derived from multiple database queries by adding noise into the response to each query. The noise hinders the attacker from revealing the distribution of data that can be used for re-identification.

**Blockchain.** One critical threat to e-health data is data tampering, which can lead to patient medical accidents. Blockchain, which provides public and distributed ledger on a peer-to-peer network, was proposed by Nakamoto in 2008 [35] to ensure data integrity by recording all verified transactions in a ledger based on a consensus algorithm.

Some studies have taken advantage of blockchains for secure data preservation [36], secure data sharing [37,38], and the access control [21,22,23,24]. Li et al. [36] designed a reliable data storage for primitiveness and verifiability of e-health data based on the blockchain while preserving privacy. The anonymity of users and the data was also considered by using cryptographic algorithms such as AES. Fan et al. [37] and Patel [38] proposed e-health data sharing systems between the heterogeneous databases of hospitals (i.e., cross-domain). Because of the lack of standard data management and data sharing policy in the conventional EMR systems, Fan et al. [37] proposed MedBlock that is applied blockchain with public and distributed ledger. In the proposed system, hospitals can upload encrypted data to MedBlock, thus a user who has the right decryption key can retrieve and verify the data anywhere at any time. Patel [38] also designed a blockchain-based image sharing system. The blocks recorded a list of images and related patients, authorized entities by the patient to access the images, and the retrieval endpoint (i.e., URL) that actually has the images. Only authorized users by the patient can access the endpoint and retrieve images stored in the hospital database.

However, blockchain is not suitable for big data, thus network location information indicating the desired resource can be used instead of recording large data.

**Steganography and watermarking.** Steganography is a technology that hides secret information within other data, such as a medical image, whereas encryption converts original data into data that is unrecognizable without the proper key for decryption. This protects secret information and conceals its existence. Furthermore, steganography is generally categorized into spatial domain techniques (i.e., such as least significant bit (LSB), embedding, and spread technique) and transform domain techniques (i.e., discrete wavelet transform (DWT) and discrete cosine transform (DCT)). Spatial domain techniques are fast but vulnerable to compression and geometric distortion such as rotation, scaling, and cropping, whereas transform domain techniques require high computational power but have resistance to compression and geometric distortions [39].

Karakış et al. [39] proposed similarity-based LSB and fuzzy-logic-based LSB that select non-sequential LSBs of image pixels to insert secret messages. The proposed LSBs are compressed and encrypted in the message preprocessing stage in a cover image. An only authenticated user who has the right key can decrypt and read the hidden message including electroencephalogram (EEG) signal, doctor’s comment, and patient information. Mantos and Maglogiannis [40] also developed a new LSB-based steganography method. The method hides patient data and integrity hashes in the region of interest (ROI) and recovery data into the region of non-interest (RONI) that is required to recover the ROI. In the method, the patient data are protected by AES, and integrity is achieved as well with the hashes of the ROI part and the hidden data. Moreover, Elhoseny et al. [27] utilized 2D DWT to hide the secret data encrypted by AES for secure medical data transmission in IoT environments. It has a high-security level by applying AES, RSA, and 2D-DWT; however, they could not be suitable for the IoT environments that are resource-constrained in terms of network bandwidth, computational power, memory capacity, and so forth.

In addition, digital watermarking is a promising security solution that provides content authentication, integrity, and credibility of medical images [41,42]. Digital watermarking in e-healthcare services embeds sensitive information such as patient identity and diagnostic details into medical images by converting the gray level of pixels without any perceptible changes to the host image [43]. However, watermarking can distort medical images; this is a critical issue because it can lead doctors to misdiagnose patients. Therefore, Turuk et al. [41] proposed a reversible watermarking scheme based on quantized DWT, and the scheme supports watermark extraction from images and restoring the original medical image. The proposed scheme can also embed multiple watermarks by means of quantization function, and recover the original medical image using a tracking key which preserves sign change of the original image’s coefficient. Moreover, a fragile watermark was proposed by Walton in 2007 [44], and it has been studied to detect medical image tampering based on the sensitivity. With the fragile watermark, image tampering can easily be detected, because even a one-bit change can affect the verification results of integrity. Shehab et al. [42] proposed a scheme with the advantages of the fragile watermarking technique. The proposed scheme particularly used singular value decomposition (SVD) with the 4 × 4 size of image blocks for tamper localization that can localize attacked pixels and regions. The scheme can also be used for recovering the tampered region with Arnold transform.

Finally, Table 3 shows a summary of studies related to e-health data.

## 4. Medical Device

With the advancement of sensors and network technologies, medical devices such as wearable devices and IMDs have been connected to networks to enable smart e-healthcare services, such as remote diagnosis and prescription. Medical devices play a role of sources that produce a huge amount of e-health data; therefore, the security of medical devices should be properly considered.

### 4.1. Overview

**Figure 4:**
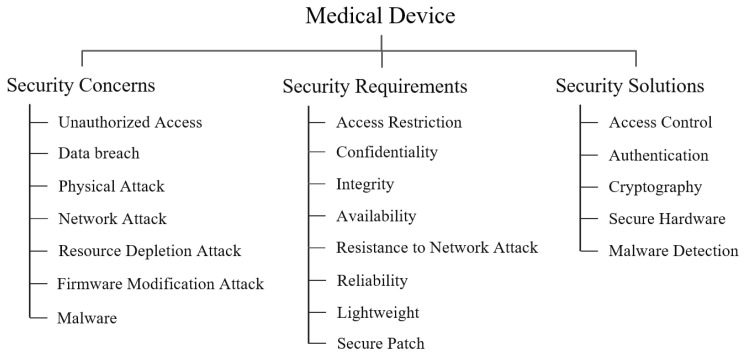
Taxonomy for security and privacy on medical device.

Figure 4 shows the taxonomy for security and privacy on medical devices. In a nutshell, physical and logical access control schemes, including proper authentication, must be adopted to prevent unauthorized access and network attacks on medical devices and cryptography should be required to protect the sensed e-health data and credentials stored in the device. In addition, secure hardware can be used to enhance the security of resource-constrained medical devices, and malware detection techniques are required since devices can be compromised by malware.

### 4.2. Security Concern, Requirement, and Solution

This section presents security concerns, requirements, and solutions for medical devices. Note that a limited number of studies are surveyed because of insufficient studies for medical devices that fulfill the selection criteria described in Section 2.1.

#### 4.2.1. Security Concern

There are several security concerns that have implications for medical devices. Since the medical devices are networked, an attacker can access the device through the network to breach e-health data, or compromise the device using malware to make it follows some malicious operations which can affect patients’ health condition. In addition, depletion attacks can consume device resources such as computing power and battery to interrupt desired operations of the device so that it cannot provide e-health data to someone who needs the data. Detailed descriptions for the security concerns that are commonly described in several studies are as follows.

**Unauthorized access.** An attacker can access medical devices by means of some security holes in the devices. Unauthorized access from an attacker or user who has a malicious purpose can cause a wide range of concerns from data breach of patients to life threats. According to Yaqoob et al. [3], various attack methodologies such as reverse engineering and communication channel exploitation (e.g., lack of encryption, authentication, and access control) were used for the unauthorized access.

**Data breach.** As described in Section 3, data including e-health data and credentials stored in medical devices can be leaked, tampered with, or deleted by unauthorized access. Protecting data stored in devices and transmitted via the Internet requires proper security solutions such as authentication, access control and cryptography. In addition, user identities should particularly be secured since the loss, theft, and disclosure of personally identifiable information accounts for one-fifth of all reported issues [45].

**Network attack.** As medical devices have been connected to medical networks and the Internet to support modern healthcare services, the network has become an entrance to medical devices [12,46]. In general, there are two types of network attack: passive and active. Passive attacks harm confidentiality by observing or copying network traffic and active attacks infringe on the integrity and availability by controlling the network traffic and modifying the messages in the traffic. Section 5 describes the network attacks in more detail.

**Physical attack.** A physical attack is one of the most representative security concerns for medical devices. Medical devices can be damaged by natural disasters or a malevolent person. In particular, someone can access or steal medical devices to capture patients’ private information.

**Resource depletion attack.** Most medical devices have limited resources such as computational power and battery life. An attacker can deplete medical device resources so that they no longer work properly. Since medical devices can directly affect patients’ health conditions, a resource depletion attack is very critical to the security of medical devices. A power-draining attack, which is a type of resource depletion attack, was demonstrated by Hei et al. [47].

**Firmware Modification attack.** This attack modifies the firmware stored in non-volatile memory that controls medical devices [3]. An attacker can inject malicious firmware into a device when it needs to be updated. By modifying or changing the original firmware, an attacker can control the medical devices as desired.

**Malware.** Malware, such as spyware, botnets and Trojans, are malicious software that can damage medical devices. Malware that controls devices are particularly critical because they can affect patients’ health condition and life [45]. Proper security solutions must check the data transmitted from other networks to detect or prevent malware injection. Since medical devices have constrained resources, a proxy could facilitate the solutions instead of the devices.

#### 4.2.2. Security Requirement

As medical devices are networked, network attacks must be considered to secure the devices and the e-health data that are generated and stored in the devices. Security requirements that are generally mentioned in the medical device studies are as follows.

**Access restriction.** Unauthorized access to e-health data must be restricted appropriately. In other words, access must be authenticated and authorized to determine whether the user who requests data has proper permissions. This requirement includes the restriction of physical access and information access.

**Confidentiality.** E-health data and the credentials of medical devices must be confidential. In general, security by obscurity and cryptography are used to protect data confidentiality; however, security by obscurity is increasingly insufficient and strong cryptography has become important [48].

**Integrity.** Protecting the integrity of firmware and software and data integrity are critical issues for medical devices since compromised firmware and software can control devices. The violation of the integrity can affect patients’ health condition and, thus, it is one of the most important security requirements.

**Availability.** In addition to data availability, medical devices must also be available any time when the owner wants to use them or for medical staff in an emergency. Several attacks such as DoS and packet flooding attacks can infringe upon the availability of medical devices, similar to data availability. Fault tolerance is the one of primary functionalities that makes medical devices work consistently even when compromised.

**Resistance to network attack.** Several network attacks such as eavesdropping, replay, and impersonation can compromise medical devices. To design secure medical devices, network attacks must be considered since the devices have been connected to both medical networks and the Internet. This requirement is also important to protect against other attacks such as resource depletion and malware. Section 5 describes network attacks and related requirements in detail.

**Reliability.** A medical device has its own purpose and intrinsic features are important to a patient’s health condition. Malfunction of medical devices due to various causes such as software bugs, malware, and security attacks could damage patients. To protect patients’ safety, medical devices must provide reliability in terms of performing their intended function.

**Lightweight.** Security solutions adopted in medical devices should be lightweight because medical devices have resource constraints. Security solutions should work with limited resources to fulfill the minimum security requirements for medical devices and the data they hold.

**Secure patch.** Firmware and software are imperfect; they have hidden flaws and vulnerabilities including zero-day vulnerabilities [46]. Therefore, medical devices must have the ability to securely patch the firmware and software when vulnerabilities are uncovered and must be able to verify whether the firmware or software is untampered. This verification is required since the firmware and software downloaded via the network can be modified to compromise devices [48].

#### 4.2.3. Security Solution

Representative security solutions to protect medical devices are authentication, access control, and cryptography. Since general medical devices are resource-constrained, the security solutions should be efficient and lightweight, and secure hardware can be used to enhance the security of the device. Detailed descriptions for the security solutions of the medical device are as follows.

**Access control.** Access control is an essential security solution to restrict unauthorized access. Access control for medical devices is crucial because it can be related to patients’ health condition and life. According to Wu et al. [10], there are two types of direct access control schemes: using a preloaded key and temporary key and an indirect access control scheme using a proxy. The direct access control schemes basically permit access by validating a key within a medical device, while the indirect access control scheme delegates the access control to a proxy server (e.g., smartphone and smartwatch) since the medical device has limited resources.

**Authentication.** Any user who accesses medical devices must be properly authenticated. In general, there are three factors in authentication schemes: ownership, knowledge and biometric.

Most medical devices authenticate a valid user based on their knowledge such as ID and password; however, biometric-based authentication schemes have recently emerged for the medical devices. Security by obscurity is not enough to secure medical devices [48]. Liu et al. [49] proposed local authentication and remote authentication for the cloud-assisted wearable devices. In the local authentication protocol, Hash-based selective disclosure mechanism and Chebyshev chaotic map are used to realize mutual authentication between a wearable device and a smartphone. After the local authentication, the cloud performs remote authentication of the device based on a yoking-proof. In addition, to make accessing the device challenging, multi-factor authentication [50], and biometric-based authentication schemes [51,52] can also be used. Zheng et al. [51] proposed a finger-to-heart (F2H) IMD authentication scheme that allows a doctor to access a patient’s device by scanning the fingerprint of the patient in an emergency. They emphasized that the proposed scheme is suitable for IMDs than ECG-based authentication scheme. Because the scheme requires only low resources because it is not required to capture or process biometric in every access. Belkhouja et al. [52] proposed a two-factor authentication scheme for IMDs using ECG signal and fingerprint.

ECG was used to authenticate medical staff in an emergency, and fingerprint was utilized as an assistance factor of the authentication.

Moreover, a lightweight and low power authentication scheme is required to solve the resource constraint problem of medical devices. For example, Halperin et al. [53] proposed a zero-power authentication method based on radio frequency (RF) power harvesting of an IMD programmer (i.e., a proxy device).

**Cryptography.** Cryptography is required to protect e-health data produced by medical devices and the devices’ credentials. In particular, strong encryption is required to protect highly sensitive e-health data [48]. Zheng et al. [54] proposed an ECG-based data encryption scheme for IMDs. The scheme used one-time pads (OTPs) generated from ECG signals as a key for encryption. In addition, the OTP-based keys are dynamically generated for each round of encryption, thus additional processes (i.e., require key distribution, storage, revocation, refreshment, and seed protection) are not required. In addition, lightweight and low-power cryptography are required for medical devices that are resource-constrained in terms of computation power, memory, and battery.

**Secure hardware.** Medical devices generally have constrained resources in terms of computing power and battery, which hinder their adoption of strong security. Therefore, secure hardware such as a hardware security module (HSM) and physical unclonable function (PUF), which would take care of security-related processes, can be used to enhance the security of medical devices. Diverse security solutions such as cryptography, authentication, and access control can be supported by secure hardware.

**Malware detection.** Malware detection techniques such as control-flow integrity verification and call stack monitoring are important for medical devices because malware remains unknown until detected [45]. The detection techniques are critical since undetected malware can consistently affect devices. In addition, hardware-based malware detection is a promising security solution because of the resource constraints of medical devices. Once the malware has been detected, it must be properly treated.

Table 4 shows a summary of the security and privacy studies for medical devices.

## 5. Medical Network

This section presents security concerns, requirements, solutions, research trends and open challenges for security and privacy in medical networks. Since modern e-health systems are based on the network, the security and privacy for the network are must be considered to design secure e-health systems. Note that the term “medical networks” used in this paper includes diverse types of networks that transmit e-health data such as IoMT and WBAN.

### 5.1. Overview

**Figure 5:**
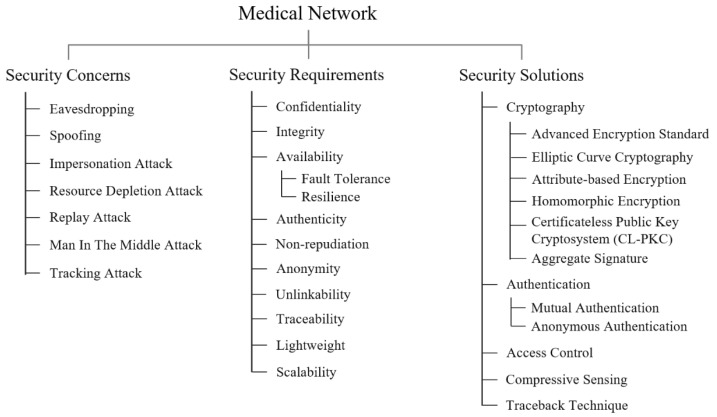
Taxonomy for security and privacy on medical network.

We classified the security and privacy studies that focused on medical networks in terms of security concerns, requirements, and solutions. Figure 5 shows a taxonomy of these studies.

In a nutshell, as shown in Figure 5, there were five security solutions for medical networks, 11 security requirements for the solutions, and eight security concerns remaining to be solved. In particular, cryptography, authentication, and access control were widely studied to provide data confidentiality, integrity, anonymity, authenticity, and non-repudiation against diverse security concerns such as eavesdropping attacks, denial of service (DoS), replay attacks, impersonation, man in the middle (MIMT) attacks, and spoofing attacks.

### 5.2. Security Concern, Requirement, and Solution

This section presents security concerns, requirements, and solutions for medical networks, such as WBANs and IoMTs, based on the medical network taxonomy.

#### 5.2.1. Security Concerns

Similar to the conventional network, there are passive attacks and active attacks in medical networks. In other words, an attacker can eavesdrop the network communications and interrupt the communications to breach e-health data which is highly sensitive information. The six general security concerns that are the goals of the recent studies are as follows.

**Eavesdropping.** An adversary can eavesdrop on the traffic of medical networks to capture useful information such as patients’ e-health data. Even though the data in the air is generally anonymized or encrypted, this attack can be one of most critical because other attacks use the data captured by the eavesdropping attack; it becomes more serious if the data has not been properly anonymized or encrypted. Spoofing. Data such as nodes, identity information, and network addresses can be forged by an attacker in medical networks [55,56]. The attacker exploits a spoofing attack to deceive legitimate users or security systems for unauthorized access or further attacks.

**Impersonation attack.** An adversary can impersonate a legitimate entity on a medical network such as a user, device, or server by eavesdropping on some network traffic. The attacker can then perform other attacks using the impersonated identity [57]. This attack can be posed by weak authentication [58].

**Resource depletion attack.** Resource depletion in medical networks is an attack that threatens to exhaust network resources such as bandwidth and traffic. Medical networks such as IoMTs and WBANs are particularly lacking in resources; therefore, this type of attack can easily hinder the availability of medical services operated in medical networks. DoS is a typical resource depletion attack.

**Replay attack.** A replay attack can be done by capturing network packets and then transmitting them instead of the legitimate sender. This attack could be performed by an attacker to make a medical device or a server unavailable or to impersonate a valid user. To avoid replay attacks, random numbers or timestamps are generally included in packets.

**Man in the middle attack.** A man in the middle (MITM) attack can be done by intercepting and controlling the network communication between the two parties (e.g., medical devices and servers). It is difficult for the victims to detect the presence of an adversary, so they should believe that communication may be modified and transmitted by an adversary. If communication is related to remote treatment and prescription, this attack becomes very critical to patients.

**Tracking attack.** An attacker can track patient locations (e.g., their workplace or home) by monitoring medical networks to discover the identity of the patient and some additional related information [13]. The attacker can track several networked devices such as smartphones, smartwatches, medical devices, and RFID tags.

#### 5.2.2. Security Requirements

Security concerns for the medical networks are similar to the conventional networks; however, the medical networks mostly transfer e-health data with the patient’s identity, which are highly sensitive. Therefore, security requirements for medical networks should be more rigorous than the conventional network [14]. In this section, the ten representative security requirements commonly mentioned in the medical network studies are presented.

**Confidentiality, integrity, and availability.** Data confidentiality, integrity, and availability were already discussed in Section 3; however, the requirements for these are more stringent when the data are transmitted via medical networks. E-health data must satisfy confidentiality and integrity since an adversary in a medical network can eavesdrop and modify the data. In addition, data availability is a prominent requirement in medical networks. Patients must be able to use their data whenever they want and medical staff must be able to use the data in a remote healthcare system, particularly in an emergency.

**Authenticity.** An adversary in medical networks can forge a message or impersonate a user. Therefore, the authenticity of each message’s origin [59] and a user’s identity [60] must be properly checked to prevent attacks on authenticity.

**Non-repudiation.** Non-repudiation denotes the ability that can assure that someone cannot deny the validity of something [61]. For example, non-repudiation could be provided for a doctor’s diagnosis in case of a medical incident [62].

**Anonymity.** Data anonymity is important; moreover, the identities of patients in medical networks must be anonymized. By making patients’ identities anonymized, an adversary who eavesdrops on network communications cannot obtain patients’ real identities.

**Unlinkability.** Even though e-health data or patient identity is anonymized, an adversary in medical networks must not be able to link captured data with a specific sender. If the data or identity of communications is linkable, the adversary may combine some data to obtain a personal health record by requesting different types of anonymized data for a person. Therefore, both anonymity and unlinkability are important in medical networks.

**Traceability.** In general, a user’s true identity must be anonymized to prevent a tracking attack for that identity by an adversary on medical networks. However, the true identity might be conditionally revealed when it is related to the adversary on the networks [60,63]. This requirement should be supported in special cases and must be carefully treated since it can also uncover a patient’s true identity.

**Lightweight.** Security solutions such as cryptography, authentication, and access control for medical networks should be lightweight since medical networks have limited resources in terms of bandwidth, traffic, and network nodes’ hardware specifications. Several studies have focused on lightweight security solutions [55,57,64,65,66], and the lightweight scheme becomes more important; however, strong security solutions that require high levels of resources are still needed to secure e-health data. Therefore, maintaining an appropriate tradeoff between efficiency and strength in security is a critical issue in medical network security.

**Scalability.** As the number of users, medical devices, and e-health data increases in medical networks, scalability for networks should be supported. Scalability is an important security requirement because it is related to availability, which is a very critical security requirement in medical domains. Based on scalability, medical services that use the networks can be continuously provided for patients.

#### 5.2.3. Security Solution

This section describes five security solutions: cryptography, authentication, access control, compressive sensing, and traceback technique. Most studies were particularly focused on cryptography to protect the patients’ data and authentication schemes to check the true identity of network entities. In addition, since medical networks are resource-constrained, the studies in this area mainly aimed at efficient and lightweight security solutions.

**Cryptography.** There have been considerable studies on security and privacy that take advantage of diverse cryptography techniques in medical networks. A brief introduction to cryptography techniques and studies is as follows.

*Advanced encryption standard.* Lounis et al. [59] applied AES, and randomly generated symmetric key (RSK) to encrypt medical data for cloud-based scalable architecture, and the architecture can securely store and shares patient’s health data in wireless sensor networks (WSNs). The authors overcame the overhead of ABE by encrypting an AES key (i.e., RSK) rather than encrypting the whole of medical data. Guo et al. [55] also adopted AES for a lightweight encryption/decryption scheme in WBANs environment. They proposed a secure and privacy-preserving framework based on multi-level trust management with opportunistic computing [67]. The opportunistic computing allows an opportunistically contacted node to assist other WBAN node’s operations when the node has not enough energy and computing power. In the framework, different privacy protection strategies were applied for user’s privacy based on the groups which have different trust levels.

*Elliptic curve cryptography.* Elliptic curve cryptography (ECC) is a form of public-key cryptography using elliptic curves over finite fields. Compared with conventional public-key cryptosystem, ECC is faster and more efficient in terms of computational time, memory capacity, and bandwidth [57]. Therefore, most studies that use public-key cryptography were based on ECC for resource-constrained medical networks. Some studies [57,60,68] adopted ECC for design efficient authentication protocols, and Omala et al. [69] proposed a secure transmission scheme based on the ECC.

**Figure 6:**
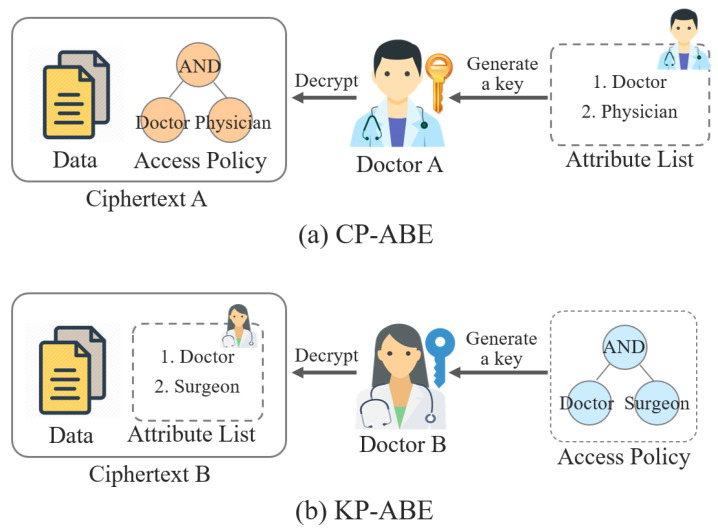
Overview of ABE schemes.

*Attribute-based encryption.* Attribute-based encryption (ABE), which is a type of public-key cryptosystem, was first proposed by Sahai and Waters in 2005 [70]. In many studies on medical network security, ABE was adopted to implement flexible and fine-grained access control systems [59,63,71] for e-health data, since the data can be encrypted based on diverse attributes such as patient name and treatment date. Conventional ABE is divided into two types: ciphertext-policy ABE (CP-ABE) and key-policy ABE (KP-ABE). The main difference between CP-ABE and KP-ABE is the position of the access policy. In CP-ABE, an access policy is encrypted with e-health data, whereas the policy is used to generate a decryption key in KP-ABE. Figure 6 shows the difference and overview of CP-ABE and KP-ABE.

In Figure 6a, Doctor A, who has a decryption key and the attributes Doctor and Physician, can decrypt the ciphertext A, which includes the access policy consisting of Doctor AND Physician. Meanwhile, in Figure 6b, Doctor B can decrypt the ciphertext B encrypted with the attributes Doctor and Surgeon if they have a decryption key generated using the access policy consisting of Doctor AND Surgeon. CP-ABE and KP-ABE are very promising cryptographic schemes for various applications; however, ABE is not suitable for resource-constrained medical networks such as WBANs and IoMTs because it requires high performance for cryptographic operations. Therefore, some methods have been proposed to overcome the resource limitations. To reduce computational overhead, Lounis et al. [59] encrypted secret keys for e-health data rather than encrypting the entire e-health data. On the other hand, Zheng et al. [71] used online/offline encryption techniques [72] to efficiently apply ABE into medical networks. In the offline phase, some cryptographic operations are performed in advance before the message to be encrypted is entered, which is required in the encryption phase. Then, based on the results of the online phase, encryption is performed in the online phase.

*Homomorphic encryption.* E-health data are highly sensitive. Even though the aggregation of e-health data could be very useful for various e-healthcare services, data confidentiality should be preserved when aggregators collect data from personal medical devices. With homomorphic encryption, e-health data collected by aggregators can be processed without decryption, thereby preserving privacy. In addition, data aggregation techniques are used to reduce the communication cost of medical networks (e.g., WBANs and IoMTs) in real-time data transmission.

Ara et al. [73] proposed a secure privacy-preserving data aggregation (SPPDA) scheme based on the bilinear ElGamal cryptosystem, which has the homomorphic property, for remote health monitoring systems.

To privately aggregate the e-health data from sensing nodes of patients, the aggregators adopt pairing-based homomorphic encryption and send the collected data to the medical server. In general, pairing operation requires high computation cost, however, this study executed heavy operations such as key generations and decryption in remote medical servers for efficiency. Huang et al. [74] collected e-health data from WBANs and transmitted the data to wireless personal area networks through WSNs by means of homomorphic encryption based on the matrix (HEBM), and Tang et al. [64] also proposed a privacy-preserving health data aggregation scheme that can securely collect health data from healthcare devices. In the study, Boneh–Goh–Nissim (BGN) cryptosystem was used which has some homomorphic features. In addition, Wang and Zhang [75] proposed a data division scheme using homomorphic encryption to prevent eavesdropping attacks in WSNs. By using the homomorphic encryption, e-health data was divided into three parts, sent to the central server separately, then merged and stored in the server after checking the integrity with the message authentication code (MAC) of the divided data. Wireless environments including WBANs and IoMTs are vulnerable to eavesdropping; however, patient’s privacy may not be fully disclosed since the data are divided.

*Certificateless public-key cryptography.* Due to the resource constraint problem in medical networks, traditional public-key infrastructure (PKI) is unsuitable for medical networks. Moreover, certificate management that needs a trusted third party, a certificate authority, is an obstacle. Therefore, an identity-based cryptosystem (IBC) [76] was proposed to remove certificates. IBC had a key escrow problem, but Al-Riyami and Paterson [77] solved this problem by proposing certificateless public-key cryptography (CL-PKC). In addition, signcryption [78], which is more efficient than a conventional sign-then-encrypt technique, has been widely adopted in WBANs.

To secure transmission between WBANs and servers, Omala et al. [69] designed an ECC-based certificateless signcryption (CLSC) scheme, and the scheme is lightweight and resistant to key escrow attack. Barbosa and Farshim (BF) [79] previously built a base scheme of this study using bilinear pairing, however, they improved the performance in terms of computation cost and energy consumption by means of ECC, and they utilized the proposed scheme to secure transmission from WBANs to a medical application provider. According to the evaluation results, the proposed scheme showed better performance in terms of energy consumption by 46% than BF’s scheme. Li et al. [80] also designed a CLSC scheme based on the identity-based signcryption (IBSC) scheme [81]. Based on CLSC, they solved key escrow and certificate management problems. Shen et al. [57] and Ji et al. [60] also adopted certificateless scheme to eliminate public key certificates in their authentication protocols.

Moreover, Zhang et al. [82] proposed a lightweight and secure device-to-device (D2D)-assisted data transmission protocol based on the CL-PKC in m-health systems. In general, there are three techniques of CL-PKC: certificateless signature, certificateless encryption, and certificate signcryption. This study particularly adopted certificateless generalized signcryption (CLGSC), which can support the three CL-PKC techniques, to provide data confidentiality, integrity, mutual authentication, contextual privacy. In addition, anonymity and unlinkability were also supported by using pseudo-identity and a random nonce value. They used D2D communications to transmit big health data collected by BAN instead of cellular networks that are already overburdened to transmit other data.

**Authentication.** Authentication is an essential security function for medical network security to authenticate unknown users or devices. In medical networks, authentication schemes/protocols have been widely studied considering various security requirements such as integrity, anonymity, unlinkability, authenticity, non-repudiation, and forward/backward secrecy to prevent several security concerns such as replay, impersonation, MITM, and spoofing attacks. According to our survey, authentication studies for medical networks generally considered efficiency and they were classified as mutual, anonymous, or certificateless authentication.

Some studies [62,83,84] designed a mutual authentication protocol. Li et al. [62] proposed a mutual authentication protocol and key agreement scheme based on Chebyshev chaotic maps and Diffie–Hellman key exchange. In the proposed medical system, only authorized doctors and medical staff can have permissions including access to patients’ health data collected from patients’ body sensors. In addition, a digital signature was utilized to provide non-repudiation for the doctor’s diagnosis. Cheng et al. [83] applied blockchain to avoid strong dependence on a trusted third party for a mutual authentication scheme. Ibrahim et al. [84] proposed a lightweight mutual authentication scheme for two-tier WBANs to ensure the originality and integrity of patient health data with anonymity between various body sensors. The proposed protocol only applied hash and XOR operations and required 480 bits memory on each WBAN nodes, and this characteristic makes the protocol is efficient for resource-constrained environments.

Certificateless authentication scheme is also researched. Shen et al. [57] presented an efficient multi-layer authentication protocol with a secure session key generation scheme and characteristics of WBANs. The proposed authentication protocols support two layers in WBANs. In the communication layer, sensors-to-personal digital assistance (PDA) group authentication protocol considering resource constraint of WBAN nodes was designed for performing between PDA and sensors. In the second layer, completely wireless environments are considered, and a non-pairing certificateless authentication protocol was designed to be used between PDA and application providers based on ECC that is an efficient scheme for WBANs. Ji et al. [60] also proposed an efficient and certificateless conditional privacy-preserving authentication scheme for WBANs based on ECC. They consisted that the traceability of real identity in anonymous environments is conditionally required because anonymity could be exploited by a malicious user. In an emergency, a trusted authority that acts as a key generation center (KGC) also can trace the real identity of a patient. In addition, to improve performance, the proposed scheme supported batch authentication which validates multiple WBAN clients at the same time.

In addition, there were authentication protocols for radio frequency identification (RFID). RFID is a promising identification technology to manage medical supplies, equipment, medications, and patients. In medical domains, RFID tags could contain sensitive information such as patients’ health data that require high security. Rahman et al. [85] proposed a privacy-preserving framework named PriSens-HSAC for RFID to support a group based anonymous authentication protocol. In order to authenticate a tag, a reader sends a challenge to a tag, and the tag responses to the reader by encrypting the challenge, identity of the tag, and a nonce with a group key. Jin et al. [68] proposed a secure ECC-based RFID mutual authentication scheme for patient medication safety. The proposed scheme consists of two phases: setup phase and authentication phase. In the setup phase, a back-end server creates public/private keys and the identity value of the tag (i.e., a random point on the elliptic curve), then the server sends the identity to the tag. Based on the setup parameters, the server and the tag can authenticate each other.

Fan et al. [65] presented a lightweight RFID medical privacy protection scheme in IoT. This study strongly depended on the proposed cross operation (i.e., the operation of bit cross) and index data table for an efficient RFID authentication scheme. However, Aghili et al. [66] identified several vulnerabilities of the authentication protocol proposed by Fan et al. [65] in terms of secret disclosure, reader impersonation, and tag traceability attack. Then, they proposed an improved mutual RFID authentication protocol, SecLAP, for secure communication and privacy protection in medical IoT. Recently, Attarian and Hashemi [86] researched an anonymity communication protocol based on blockchain and user datagram protocol (UDP) in mHealth environments. Their protocol was specifically designed to protect the data security and privacy of clients’ identities.

**Access control.** There were various access control studies in different target domains; therefore, this section specifically presents access control schemes focusing on medical networks. Lounis et al. [59] and Yang et al. [63] proposed a fine-grained access control framework based on ABE for the medical networks (i.e., WSNs and IoT). Lounis et al. [59] proposed an efficient fine-grained access control that supports complex and dynamic security policies using CP-ABE, and Yang et al. [63] also proposed a privacy-preserving e-healthcare system that provides fine-grained access control and flexible access policy update.

Since user identity is very sensitive information in medical networks, Li et al. [80] proposed an anonymous access control model based on the proposed certificateless signcryption (CLSC) scheme that is cost-effective for WBANs. Their proposed access control model has advantages that it does not have a key escrow problem and public key certificates that is required to be managed.

In addition, there was a study applied break-the-glass concept for the emergent situation. Maw et al. [87] proposed a flexible access control model, break-the-glass access control (BTG-AC), for medical data in wireless medical sensor networks. The model was mainly considered to solve the conflict between data privacy and availability using break-the-glass (BTG) concept. Unlike the conventional BTG-RBAC model, the proposed BTG-AC used BTG policy only in emergency situations with Ponder2 policy package, and it is designed to be lightweight for WSNs.

**Compressive sensing.** By using compressive sensing (CS), the effect of full sampling can be achieved with just a few sampling points [88]. Since the medical networks are resource-constrained, CS can be adopted to reduce communication costs while maintaining data confidentiality.

Peng et al. [58] proposed a secure and energy-efficient e-health data transmission system based on chaotic CS, which is energy-efficient and also has an encryption performance for the medical networks. Since conventional CS uses measurement matrices for both senders and receivers, they need huge storage space. Therefore, chaotic CS was adopted, which only requires partial parameters for matrix generation such as the chaotic parameter, initiation value, sampling initial position and distance as a key, to save the storage space. In addition, it is more secure than traditional CS techniques because of the sensitivity of chaos.

**Traceback technique.** A DDoS attack is a critical attack against the medical networks since it depletes the networks’ limited resources and thus hinders the transmission reliability of patients’ e-health data. Therefore, DDoS detection techniques represent an important research subject in this domain. According to Latif et al. [56], the probabilistic packet marking (PPM) traceback technique is widely used in IP-based networks to detect the source of a DDoS; however, it cannot be directly applied to a resource-constrained WBAN environment because of its high convergence time and overhead on sensor nodes in WBAN. Therefore, Latif et al. [56] presented a novel approach, efficient traceback technique (ETT), based on Dynamic Probabilistic Packet Marking (DPPM). In other words, they utilized variable marking probability based on the packet’s traveling distance with DPPM label in the MAC Protocol Data Unit (MPDU) to the target node.

Finally, Table 5 summarizes the study analysis.

## 6. Edge, Fog, and Cloud

Recently, conventional healthcare systems have been combined with diverse technologies, such as big data, IoMT and WBAN, to provide more advanced e-health services. Cloud computing is a promising computing paradigm that is being used in medical research areas since it provides various advantages such as cost efficiency, scalability, availability, and flexibility. In addition, edge and fog computing have been studied to support time-sensitive medical operations. This section presents security concerns, requirements, solutions, research trends, and open challenges in edge, fog, and cloud computing.

### 6.1. Overview

**Figure 7:**
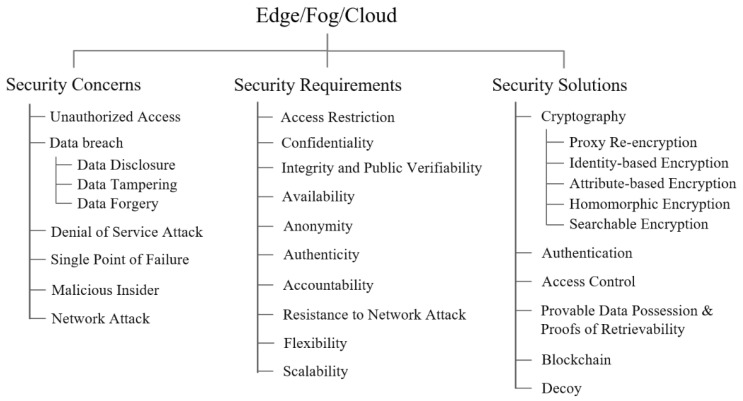
Taxonomy for security and privacy on edge, fog, cloud computing.

Figure 7 shows the taxonomy for the security and privacy studies in edge, fog, and cloud computing. In a nutshell, the security and privacy studies that deploy edge, fog, and cloud computing generally applied cryptography, authentication, and access control to ensure various security requirements such as data confidentiality, integrity, availability, and public verifiability, that solve several security concerns such as unauthorized access, data breach, and single point of failure (SPoF). In particular, security solutions for cryptography, authentication, and access control have been studied to protect e-health data in the cloud since the cloud has mainly been utilized as secure data storage. Furthermore, provable data possession (PDP) and proofs of retrievability (PoR) that allow patients to verify the integrity and availability of outsourced e-health data have been studied since the edge, fog, and cloud cannot be fully trusted.

### 6.2. Security Concern, Requirement, and Solution

These computing paradigms improve accessibility, usability, and manageability for e-health data, meanwhile, responsibility for ensuring strong security and privacy of e-health data becomes increased because security attacks on the edge, fog, and cloud can affect huge number of patients. Therefore, security concerns, requirements, and solutions for the edge, fog, cloud computing for e-health data should be rigorously identified and discussed to securely protect the big e-health data.

#### 6.2.1. Security Concern

Edge, fog, and cloud process and store diverse and various e-health data; hence, data breach is one of the most critical security concerns in the edge, fog, and cloud computing. Six security concerns that are generally mentioned in surveyed studies on the edge, fog, and cloud computing are as follows.

**Unauthorized access.** In addition to medical devices and networks, a secure storage that stores and processes e-health data based on edge, fog, cloud computing needs to restrict unauthorized access to the storage. Since the edge, fog and cloud are data aggregation points, robust access control is highly required to protect big e-health data by restricting unauthorized access.

**Data breach.** Data breaches, including data disclosure, tampering and forgery, present critical threats to e-health data in edge, fog, and cloud environments. Section 3 describes data breaches in more detail.

**Denial of service attack.** The cloud centrally provides various medical services; therefore, DoS attacks are a serious threat to the cloud, edge, and fog that can halt medical services. If medical services stop working, this can directly affect people’s lives.

**Single point of failure.** The major characteristic of the cloud environment is centralization. Although there are some advantages to centralization, SPoF has emerged as a main drawback.

**Malicious insider.** Even if all security solutions are well-designed and properly applied to the edge, fog, and cloud environments, malicious insiders who have the correct permissions can abuse or misuse systems for malicious purposes.

**Network attack.** The network is an essential component for using the edge, fog, and cloud; therefore, network attacks such as eavesdropping, replay, and impersonation can be used to attack the edge, fog, and cloud environments. For example, an adversary can eavesdrop on network traffic in those environments to capture useful information (e.g., users’ e-health and authentication data) for further attacks on the edge, fog and cloud.

#### 6.2.2. Security Requirement

Data security is particularly important security requirement in edge, fog, and cloud computing and a user should be able to check the data status publicly because the edge, fog, and cloud are generally managed by semi-trusted party which cannot be fully trusted. In addition, efficiency and lightness are less important compared with other domains such as the medical device and network since the edge, fog and cloud have sufficient resources. More specifically, there are ten common security requirements that can solve the security concerns of the edge, fog and cloud computing.

**Confidentiality.** As described in Section 3, data confidentiality is required in the edge, fog, and cloud environments. In particular, data confidentiality is more critical in the cloud than medical devices or networks because various and diverse e-health data are collected extensively from patients over long periods of time. If e-health data are disclosed when transmitted via medical networks, it shows very limited health information; however, if the data stored in the cloud is exposed, it can show the medical history of some or all patients in the cloud. Therefore, data confidentiality in the cloud, which is secure data storage, is particularly important compared with other target domains.

**Integrity and public verifiability.** Data integrity must be satisfied not only when transmitted over medical networks but also within the edge, fog, and cloud. The data stored on personal medical devices can easily be checked by the patients; however, outsourced data in the edge, fog, and cloud environments are difficult to check despite the patient being the data owner since the environments are managed by a service provider. Therefore, the patients should be able to check the integrity of the outsourced data stored in the edge, fog, and cloud environments.

**Availability.** E-health data in the edge, fog, and cloud environments must be available when the patient who owns are the data owner wants to use it. To this end, the edge, fog, and cloud environments must provide availability.

**Anonymity.** Anonymity of original e-health data in the cloud is achieved by means of encryption. However, the data must have been anonymized if it is required to be analyzed or shared for some reasons such as medical research.

**Authenticity.** To secure e-health data, both data authenticity and identity authenticity of a user must be provided. In particular, the authenticity of a user is required to authenticate them.

**Accountability.** Since e-health data are highly sensitive, the data processed and stored in edge, fog, and cloud environments should be accountable.

**Resistance to network attack.** Network attacks must be considered to secure communication between clients (e.g., users and devices) and edge, fog, and cloud servers. In other words, the edge, fog, and cloud servers must properly authenticate and authorize users and medical devices to protect against network attacks such as eavesdropping, replay, and impersonation.

**Flexibility.** There are diverse clients in various environments that use the services provided by the edge, fog, and cloud environments; therefore, the edge, fog, and cloud environments should flexibly accommodate different environments and their various requirements.

**Scalability.** As the data are increased and diversified, cloud storage must be scalable for the large volume of big e-health data. In addition, security solutions should provide scalability as clients such as medical devices and users have recently increased.

#### 6.2.3. Security Solution

Existing strong security solutions can be adopted in edge, fog, and cloud computing based on the sufficient resources; therefore, studies in this research area focused on useful functionalities rather than efficient and lightweight security solutions. Six security solutions that were adopted by the surveyed studies to secure e-health data in the edge, fog, and cloud computing are as follows.

**Cryptography.** Cryptography is an essential security solution that has been used in across entire medical domains as well as in edge, fog, and cloud computing. Useful cryptographic schemes for the edge, fog, and cloud computing are as follows.

**Figure 8:**
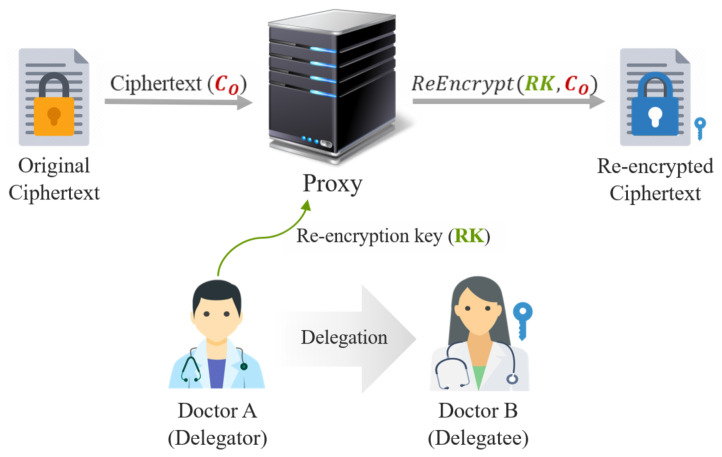
Overview of proxy re-encryption.

*Proxy re-encryption.* Permissions that allow a user to access e-health data could be changed according to the situations of a patient or medical staff. For example, if a patient’s family doctor has changed, the access permission for the patient’s e-health data must be transferred from the former doctor to the new doctor. In this context, proxy re-encryption (PRE), which was first introduced by Blaze et al. in 1998 [89], can be used as seen in Figure 8.

PRE enables a proxy to generate new ciphertext that can be decrypted by the new doctor’s private key. The re-encrypted ciphertext is encrypted using the re-encryption key generated by the delegator (i.e., the former family doctor). PRE makes the delegation more easy, secure, and private because the re-encryption operates without any decryption of the ciphertext. There are two variations of PRE, unidirectional PRE and bidirectional PRE. Bidirectional PRE schemes have the advantage that they can convert ciphertext several times; however, this may cause a data breach because of the additional re-encryption capability. Therefore, since e-health data are critical information, the unidirectional PRE scheme that re-encrypts a ciphertext once is more suitable. In the medical/healthcare research area, an identity-based proxy re-encryption (IBPRE) scheme that was first proposed by Green and Ateniese in 2007 [90] was widely adopted since the identity-based encryption (IBE) scheme can help simplify certificate management.

*Identity-based encryption.* Public-key cryptography randomly generates the public key for a user. However, an IBE scheme, which is one type of public-key cryptography, generates the public key with a user’s identity information, for example, email address. Therefore, a sender who knows the receiver’s identity information can encrypt some messages without exchanging the receiver’s public key (i.e., public-key infrastructure; PKI). The concept of IBE was first introduced by Shamir in 1984 [76]; however, the first practical IBE scheme was proposed by Boneh and Franklink in 2001 [91].

With the identity-based cryptographic concept, Wang et al. [92] proposed a new IBE scheme and a new identity-based proxy re-encryption (IBPRE) scheme and adopted the proposed identity-based cryptographic techniques into an e-health cloud system to secure e-health data. In the proposed scheme, some randomness is added to the private key to resist an adversary who compromises the private key for information of the master key. They showed the advantages of IBE that authenticates public key implicitly and simplifies the certificate management. In the proposed system, the cloud acted as secure storage and medical service provider that supports the proposed encryption scheme.

*Attribute-based Encryption.* IBE generates a public key with a user’s identity information, whereas the private key or ciphertext in ABE is generated by attributes. ABE has been considered a more flexible encryption scheme than IBE schemes since the key can be generated using diverse attributes (e.g., subject, resource, action, and environmental attributes) including identity information. In general, CP-ABE has been widely used in cloud environments to provide secure data sharing because it is much more flexible and suitable for general applications [93].

There were several CP-ABE studies [93,94,95,96,97] to protect e-health data. Wang et al. [93] proposed an efficient file hierarchy CP-ABE (FH-CP-ABE) scheme in cloud computing since the existing CP-ABE has not considered the hierarchy structure of shared files. In the scheme, the hierarchical files are encrypted with an integrated access structure to efficiently reduce storage and time cost for encryption. On the other hand, Eom et al. [94] focused on the patient-centric CP-ABE scheme. They proposed a new CP-ABE scheme, patient-controlled ABE (PC-ABE), which enables patients to control access to their own e-health data. In PC-ABE, the decryption key for encrypted e-health data was generated based on a patient’s private key and attributes of the parties that want to access the data. Since the decryption key is not generated without the patient’s private key, the patient can control the access to the patient’s data consequently. In addition, Liu et al. [95] and Rao [96] proposed e-health data sharing scheme using CP-ABE signcryption (CP-ABSC). Liu et al. [95] proposed a CP-ABSC scheme for PHR system in cloud computing based on CP-ABE and attribute-based signature (ABS) which enables a patient to sign e-health data of the patient with the patient’s private key if the patient has proper a set of attributes for the data. The CP-ABSC is a promising cryptographic technology for fine-grained access control to share e-health data in cloud computing; however, Rao [96] claimed that the Liu et al.’s scheme cannot provide confidentiality because they did not adopt the standard Signcryption techniques (i.e., encrypt-then-sign and sign-then-encrypt). Therefore, Rao proposed a new CP-ABSC scheme based on previous studies [97,98], which is more secure and efficient. The proposed scheme also can provide signcryptor (e.g., a patient) privacy and public verifiability, which are important security requirements of e-health systems in cloud computing.

*Homomorphic encryption.* Homomorphic encryption is a promising cryptographic scheme in edge, fog, and cloud computing as well as medical networks, which need to securely collect e-health data with privacy preservation. Raisaro et al. [99] proposed MedCo which enables a group of medical service providers to federate and protect the e-health data for secure sharing using the homomorphic encryption scheme in a hybrid environment that includes central and decentral environments. In other words, the proposed framework, MedCo, allows the multiple sites that store e-health data to share their data by securely querying the data to the distributed sites without sharing their databases. It also provides differential privacy by adding dummy records into patients’ e-health data. Moreover, Alabdulatif et al. [100] adopted edge computing to aggregate and analyze the large-scale bio-signal data in real-time. In the proposed edge of things (EoT) framework, fully homomorphic encryption was performed in the edge IoT gateway, located between medical devices and the cloud, to protect sensitive e-health data including patients’ privacy.

**Figure 9:**
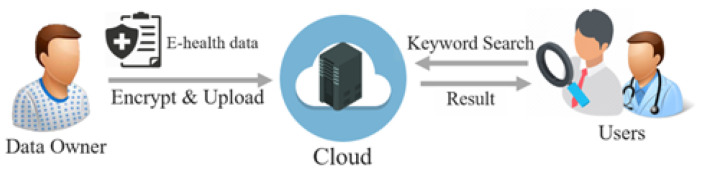
Overview of searchable encryption.

*Searchable encryption.* According to Zhang et al. [101], outsourcing e-health data and data searching services to the cloud has been a promising trend since the cloud is usually employed as data storage. In this regard, searchable encryption (SE), which was first introduced by Song et al. [102] in 2000, can be used to share encrypted e-health data in the cloud. The SE, which is a cryptographic primitive, encrypts e-health data to be keyword-searchable over encrypted data as described in Figure 9.

In a nutshell, if a patient (i.e., the data owner) first encrypts their e-health data to be searchable and uploads that encrypted data to the cloud, users (e.g., doctors and researchers) can then query the encrypted e-health data using desired keywords. Yang et al. [103], Xu et al. [104], and Chen et al. [105] adopted searchable encryption to share e-health data in the cloud environments. Yang et al. [103] proposed a new cryptographic primitive, conjunctive keyword search, with a proxy re-encryption function enabled by a designated tester (i.e., a server that can execute equality test function) and timing. Based on the proposed time-limited SE scheme, a patient can delegate access permissions to desired people so that they can search over the patient’s e-health data for a limited time. In addition, the time period to search and decrypt the patient’s data can be controlled, and the permissions are automatically revoked after the time period. Xu et al. [104] also proposed a privacy-preserving e-health data sharing scheme using SE with keyword range search and multiple keyword search. Moreover, the encrypted data can be searched by comparing different numeric types based on the proposed equality test function. In the proposed scheme, e-health data and keyword files for the data were encrypted using a symmetric key, and homomorphic encryption was used to protect the privacy of keyword in the equality test phase. Chen et al. [105] designed blockchain-based searchable encryption for e-health data sharing. They stored the indices for the data in blockchain as the form of complex logic expressions (e.g., “gender”: “male”) to make a user can use the indices for searching specific e-health data. Since the proposed scheme utilizes blockchain, it provides integrity, anti-tampering, and accountability. In addition, Yao et al. [106] proposed a multi-source order-preserving symmetric encryption (MOPSE) scheme. Compared with other searchable encryption schemes, the proposed scheme enables a data owner to efficiently query over multiple data providers’ encrypted e-health data. To this end, the cloud merges multiple encrypted indices from different data providers of the same data owner.

**Authentication.** Authentication is an indispensable security solution to prevent attackers and malicious users from accessing the data in the edge, fog, and cloud computing. The authentication becomes more important in cloud environments because the cloud stores patients’ big e-health data, which can show the patients’ medical history. There have been authentication studies focusing on mutual [107,108,109,110], anonymous [111] and traceable authentication [112].

First, mutual authentication schemes [107,108,109,110] were proposed for the edge, fog, and cloud environments. Li et al. [107] proposed a cloud-assisted mutual authentication scheme for telecare medical information systems (TMIS) by enhancing Mohit et al.’s authentication scheme [113] to be more secure and support anonymity using a dynamic pseudo-random nonce. In addition, Liu et al. proposed a novel privacy-preserving mutual authentication (NPMA) [108] and a blockchain-based privacy-preserving mutual authentication (MBPA) [109] for TMIS environments. The NPMA was designed for secure remote user authentication in the mobile edge-cloud network, which medical services are distributed in the most logical, nearby, and efficient place of the network [108]. In addition to the mutual authentication, the NPMA also provided anonymity of a patient and edge-cloud server and data confidentiality using anonyms and certificateless cryptography, respectively. On the other hand, in [109], a privacy-preserving mutual authentication was proposed for mobile medical cloud architecture based on blockchain to prevent data breach. They stored and managed the encrypted e-health data in a blockchain cloud. Each blockchain node shares the secret value for authentication. Especially, their sharing process is conducted without key negotiation rounds. Therefore, it only needs low computational cost between terminal and node rather than the traditional blockchain model. Last but not least, there was a mutual authentication study [110] designed for wearable devices using hybrid computing that consists of edge and cloud. In particular, mutual authentication was performed using the space-aware edge computing for allowing users to access the local services in a hospital.

Moreover, Mehmood et al. [111] proposed an anonymous authentication scheme based on cloud to provide complete privacy and anonymity to a user from the adversaries and the authentication server by utilizing a rotating group signature scheme based on ECC. In a group, all members share an expiration date and each of them updates their keys periodically to prevent the traceability. They also added an extra layer to provide anonymity on the network level by utilizing TOR. This can prevent traffic analysis attacks from an eavesdropper. Meanwhile, Liu et al. [112] proposed a traceable authentication protocol. They protected the privacy of patients and anonymity by means of randomized pseudonyms. The real identity of patients can also be extracted from the pseudonyms by the authentication server. The proposed authentication scheme is useful for resource-constrained mobile devices because it consumes low communication cost and energy.

**Access control.** Access control is another indispensable security solution with authentication for edge, fog, and cloud computing. Among various access control models, most studies were based on the ABE scheme to realize a fine-grained access control model [114,115,116,117,118] without a situation-based access control model [119]. In addition, there were access policy studies that focused on privacy preservation for access policy [120], dynamic access policy transformation [121], and updating access policies [122].

Since access control studies [114,115,116,117,118] are generally based on CP-ABE to provide a fine-grained access control model, the fundamental access control mechanism remains the same. E-health data are encrypted with a desired access policy using attributes and only authorized users who have proper attributes for the corresponding data’s access policy can access it. However, there are some differences in the details among the studies. Each study has an additional security solution (i.e., trust evaluation [114], dynamic auditing [115], online/offline CP-ABE [117], and unified access policy [118]) or a specific purpose (i.e., supporting multiple cloud servers [116]). The five access control studies are described in more detail below.

First, Yan et al. [114] proposed a flexible access control scheme based on ABE. Unlike the other ABE-based access control schemes, they adopted context-aware trust and reputation evaluation into the flexible access control scheme to support various data usage scenarios, for example, cloud data sharing with others. For example, data access can directly be determined by the data owner or reputation centers in an indirect way in case of the data owner is not available or cannot make an access decision. If a user has an adequate reputation, the reputation centers apply PRE to make a new ciphertext that the user can decrypt based on the pre-defined data owner’s access policy.

Second, Yeh et al. [115] proposed a cloud-based fine-grained access control framework. They controlled access using CP-ABE which enables a data owner to delegate access permissions to others by defining access policy. If a user has proper attributes for the access policy, a new ciphertext for the user is generated using PRE to make only authorized users can use the data. In addition, the proposed framework is suitable for resource-constrained IoT devices because only symmetric key encryption is used to encrypt the data when it is uploaded to the cloud. Dynamic data auditing was also used to verify data integrity using Merkle hash tree (MHT), which is a binary tree of hashes. Since a parent node’s hash is generated using the child nodes’ hashes, fast and efficient verifying integrity of e-health data can be done by checking a parent node’s hash.

Third, Roy et al. [116] designed a fine-grained access control for multi-server along with mutual authentication of users in mobile could computing environment. The proposed scheme guaranteed a low communication cost and lightweight authentication procedure because of no involvement of a registration server. It is also suitable for resource-constrained devices by mostly utilizing one-way hash function and bitwise XOR operations.

Fourth, Liu et al. [117] proposed a fine-grained access control scheme; however, they adopted online/offline CP-ABE to make resource-constrained devices in mobile cloud computing perform fine-grained data sharing. Based on the online/offline cryptography, a data owner can generate offline ciphertext before the data and access policy to be encrypted are known. The offline ciphertext which consumed a majority of computing power is then used to assemble the final ciphertext when the data and access policy are known.

Fifth, Li et al. [118] proposed a new ABE scheme for fine-grained access control framework based on unified access policy generated from multiple access policies of patients’ various e-health data. The proposed scheme improves the efficiency of encryption and decryption by combining encryption of different patients’ data that share common access policy to eliminate repetitive processes.

In addition to the ABE-based fine-grained access control schemes, Gope et al. [119] designed an access control model that can cover diverse situations including break-the-glass (i.e., emergency case) without compromising security to share e-health data based on RBAC and mandatory access control (MAC) policy. They specifically argued the access control model for e-health data should not compromise security even in an emergency because a user can misuse the break-the-glass situation for malicious purposes. To this end, this study considered the situations into the access control mechanism by proposing a situation controller that measures a patient’s situation according to the pre-defined situation types (i.e., normal, critical, emergency, and super emergency) so that it can control access depending on the situations.

Furthermore, three studies have focused on access policy in terms of privacy [120], transformation [121], and updating [122]. Ying et al. [120] designed a concealing algorithm of access policy, that can also recover hidden attributes to provide privacy regarding the access policy. To hide the access policy, they used a linear secret sharing scheme (LSSS) and proposed an element filter, Attribute Cuckoo Filter (ACF), to match whether given attributes are in the anonymized access policy. Rezaeibagha et al. [121] proposed a secure and privacy-preserving e-health data sharing scheme in hybrid cloud computing environments by transforming access policy from a private cloud to a public cloud. For the transformation, attribute-based proxy re-encryption was used. Lastly, Ying et al. [122] proposed a method to update access policy in the outsourced ciphertext of e-health data in cloud computing. Conventional CP-ABE needs to re-encrypt entire e-health data when an access policy for the data is changed, whereas the proposed scheme changes only a part of the ciphertext using LSSS if an access policy is updated.

Last but not least, Wang et al. [123] and Saha et al. [124] proposed fog computing-enabled access control schemes to protect e-health data. In more specific, Wang et al. [123] used an access controller that controls access based on the task types and pre-defined privacy levels, and Saha et al. [124] controlled access using identity token generated using the ABE scheme. They employed fog computing to reduce communication costs and response time between medical devices and the server.

**Figure 10:**
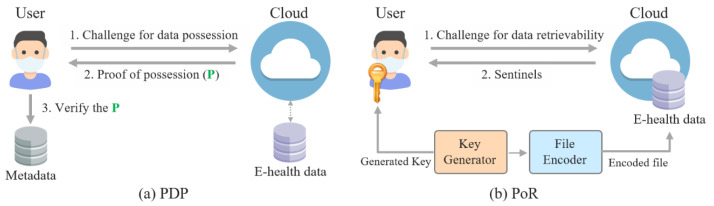
Overview of PDP and PoR.

**Provable data possession and proofs of retrievability.** Cloud storage is a semi-trust model, that is, an honest-but-curious model, so some security concerns have emerged for cloud computing. Generally, a user who outsources e-health data to the cloud cannot be aware of the data’s status. In other words, data stored in the cloud can be altered or deleted without the data owner’s consent. To solve the problem of public verifiability, provable data possession (PDP), which is a technique for checking the integrity of outsourced data, and proofs of retrievability (PoR), which is a technique that allows the owner to check the retrievability of the data, have been studied. Figure 10 shows a brief overview of PDP and PoR.

In PDP (Figure 10a), a user requests a challenge to the cloud to check the integrity of their e-health data. The cloud that received the challenge request then computes the proof of possession (P) and sends this to the user. Finally, the user who requested the P can verify the integrity of their e-health data by comparing the P with the metadata that the user stored locally when they outsourced their data to the cloud [125]. PDP is an efficient scheme to verify the integrity of outsourced data, because the user does not need to store or verify all of their data. On the other hand, PoR (Figure 10b) encrypts data and randomly embeds a set of randomly valued check blocks (i.e., sentinels). The user challenges the cloud by specifying the positions of a set of sentinels and requesting that the cloud respond to the sentinel values [126]. Based on the sentinels, the user can check the availability and integrity of the outsourced e-health data without downloading all of their data.

There are studies that propose a scheme that supports public verifiability for the outsourced data in cloud-based on PDP and/or PoR. Wang et al. [127] proposed an identity-based data outsourcing (IBDO) to provide integrity and comprehensive auditing. In the scheme, a user or an authorized proxy can outsource data in the cloud with their identities. This is efficient for multi-user environments because it does not depend on the complex cryptographic certificates to identify the clients. In addition, the origin, type, and consistency of the outsourced data can be publicly verified using the proposed scheme. Fan et al. [128] then proposed a privacy-preserving identity-based auditing scheme. This scheme enables users to share e-health data with others while it keeps the private information invisible to the cloud and others including malicious cloud manager who has high privileges. Lastly, Shi et al. [129] proposed a certificateless provable data possession (CL-PDP) scheme that provides public verifiability and complete anonymity. In particular, the proposed scheme can prevent the key escrow problem since it is based on certificateless public-key cryptography, and it is efficient because it eliminates the bilinear pairing operations which need high computational cost.

**Blockchain** A blockchain ensures data integrity and accountability by recording every transaction in a distributed ledger; this has been widely adopted to securely store and share e-health data. To provide a secure sharing scheme, studies have combined a blockchain with other security solutions, that is, access control [130], searchable encryption [131], ECC [132], and Tor [133]. Details on these studies follow below.

Nguyen et al. [130] proposed a sharing framework for e-health data in mobile cloud computing by combining blockchain and decentralized interplanetary file system (IPFS) which is a solution to realize a file sharing platform in blockchain [134]. Especially, they designed an access control mechanism using smart contracts of blockchain to securely share e-health data. However, data confidentiality may not be ensured since EHR manager where manages the encryption and decryption keys of stored e-health data cannot be fully trusted. Then, Wang et al. [131] proposed a blockchain-based privacy-preserving e-health data sharing scheme using searchable encryption and proxy re-encryption. By using the proposed scheme, a user can search required data then receive the data under the owner’s authorization. In the study, the cloud is used to store ciphertext of e-health data and re-encrypt the ciphertext for sharing, and blockchain is used to store keyword ciphertext required to search and share the data. This scheme however cannot fully ensure the owner’s data ownership because of the data provider that uploads the data to the cloud server instead of the owner. Therefore, Omar et al. [132] proposed a user-centric e-health data management system that a user has full ownership of the data based on ECC. In other words, only the data owner can control access to the data since the owner manages the encryption key. Similarly, Rahman et al. [133] proposed a blockchain-based secure therapy framework that provides e-health data integrity, privacy, ownership, and sharing. However, compared with other works, they employed mobile edge computing (MEC) and Tor. The framework reduced network latency by means of MEC and supported anonymity using the Tor.

**Decoy.** A decoy technique, also known as a honeypot, can be used to lure intruders. If an intruder touches a decoy, it is closely monitored so that a security manager can detect the intrusion and prevent subsequent attacks. A good decoy should provide detectability, conspicuousness, believability, enticement, differentiability, and non-interference [135]. The decoy should first be easily detectable and accessible, and then seem authentic and attractive to attackers; at the same time, it should be differentiable and non-interfering to ensure that naive users do not use it. In a real scenario, a decoy can be e-health data in the cloud to detect intrusions and prevent attacks. Al Hamid et al. [136] proposed a security model utilizing a decoy technique to protect big medical data in the cloud using fog computing. In the proposed security model, the fog computing facility in front of the cloud generates a decoy e-health data then shows it to an attacker who accesses the system.

Table 6 shows a summary of the security and privacy studies on edge, fog, and cloud computing.

## 7. Research Trend and Open Challenge

We reviewed recent security and privacy studies for the modern e-health systems in terms of data, device, network, and edge/fog/cloud computing. Based on the review, we identified recent research trends and open challenges for each component of the e-health systems. Therefore, we discuss the research trends and challenges in this section.

### 7.1. E-Health Data

Recent studies focused on designing a security solution that can protect the data. Most studies adopted cryptography and anonymization techniques for data confidentiality, integrity, anonymity, and secure sharing of e-health data. In addition, they proposed efficient security solutions to enhance the security and privacy of conventional e-health systems. Detailed descriptions of the research trends are as follows.

**Fast and efficient encryption scheme with high security.** Data confidentiality is the most important security requirement in the medical/healthcare research areas. Traditional cryptosystems such as AES and RSA have been widely utilized to design secure e-health systems; however, a faster and more efficient encryption/decryption scheme is particularly required when dealing with large volumes of e-health data. Since e-health data are highly sensitive, maintaining a tradeoff between the strength of encryption is a crucial issue. For example, several studies researched faster and more efficient cryptographic primitives or algorithms for medical image security [27,28,29,75].

**Securing e-health data with a blockchain.** E-health data can be lost, tampered, and deleted. A blockchain with a public and distributed ledger becomes a promising technology to secure e-health data since it records all transactions related to the data. In general, blockchains provide e-health data integrity with transparency, auditability, and accountability; however, authentication, access control, and other security applications such as secure data sharing have been studied based on smart contracts, which is a small program on a blockchain. In addition, studies have proposed efficient schemes to reduce blockchain transaction fees.

**Privacy-preserving sharing of e-health data with data anonymization.** Sharing e-health data is an emerging trend for several purposes such as remote care of individuals and studying big e-health data. To preserve privacy while sharing e-health data, data anonymization models such as k-anonymity, l-diversity, t-closeness, and differential privacy have been widely adopted in medical and healthcare research areas. Regarding this research topic, Zhang et al. [137] specifically studied on the security and privacy requirements and risks of medical data sharing based on blockchain.

Various studies for security and privacy of e-health data have been conducted; however, there are still some open challenges. In particular, re-identification prevention of anonymized data is an important research area since anonymized data can be re-identified, and data anonymization is the essential technique when e-health data should be shared. The detailed open challenges are as follows.

**More efficient and faster cryptosystem.** Though fast and efficient cryptosystems have been studied, more efficient and faster cryptosystems will be required as e-health data has become diversified and increased with emerging smart healthcare devices and services. Servers and aggregators of the e-health systems in particular need efficient, fast, and lightweight cryptosystems to provide data confidentiality with high scalability even in resource-constrained environments such as WBANs and IoMTs. In addition, considering medical imaging may become important because the medical imaging process accounts for 90% of all medical information processes [39].

**Resistance to re-identification.** Data anonymization is an essential technique to preserve privacy when e-health data are shared with someone. However, existing data anonymization techniques could be broken by some re-identification attacks. For example, Rocher et al. [138] recently proposed a model that can precisely estimate the re-identification likelihood of a specific person, even in an incomplete dataset. Data anonymization techniques will be evaluated on diverse datasets and attacks to prove that techniques can provide complete privacy preservation under any circumstances.

**Emergent access to patient’s data.** Access control for e-health data is an indispensable security solution to prevent unauthorized access to patients’ e-health data. Only authenticated and authorized users should be able to access the data by means of access control. However, medical staff may need to access patients’ data in an emergency, despite the data being highly confidential and the staff normally lacking access permission. This functionality is prominent since it can be directly related to a patient’s health condition and life in an emergency.

### 7.2. Medical Device

Conventional security solutions cannot be applied to medical devices because of the limited resources. Therefore, recent research trends for the security and privacy of medical devices are focused on efficient security solutions or a method that can alleviate the resource constraint problem. Three research trends for medical devices are as follows.

**Online authentication.** Online authentication is required when a doctor need to remotely access and monitor a medical device in an emergency situation. A secure channel should be established for the online authentication because it needs to access the device over the Internet. However, this scheme has the disadvantage that the Internet must be connected for authentication, which may not always be available [10].

**Proxy-based security.** A proxy server that supports security capabilities such as cryptography, authentication, and access control can be used between medical devices and external devices because medical devices lack the resources. Security solutions, such as IMD-Shield and IMDGuard, enhanced the security of existing medical devices in the middle of the communications [14,139,140] based on the proxy.

**Low-power and zero-power security solutions.** Security is not an essential part of the function of medical devices; however, it must be considered to secure medical devices and their e-health data. The problem is that security solutions have high energy demands for devices. Therefore, low-power and zero-power security solutions were studied to resolve the resource constraint problem of medical devices [10]. Based on the low-power or zero-power security solutions, a medical device can work securely for longer.

According to our survey, there are insufficient studies for the security and privacy of medical devices; therefore, huge effort to research the security and privacy for the modern medical devices is required in the near future. The open challenges are as follows.

**Resource constraint of medical devices.** Medical devices have limited resources such as low computing power, battery, and memory capacity; therefore, security solutions for medical devices should be designed with consideration of their constrained resources. To provide minimum security requirements for medical devices, efficient and lightweight security solutions for cryptographic primitives, encryption algorithms, authentication, and access control must be studied. As the demand for medical devices increases, efficient and lightweight security solutions for medical devices will become more important.

**Security and privacy by design.** Networked medical devices have been newly developed to support modern e-healthcare services. Therefore, the security concerns and requirements for medical devices have not been sufficiently studied and conventional security solutions are not suitable for medical devices because of their characteristics. To improve security and reliability, security and privacy by design are required to identify and adopt optimal security solutions for medical devices and their sensitive e-health data by studying their major security concerns and requirements.

**Trust management.** Medical devices that sense a patient’s health condition should manage the trust. It is important to provide a certain level of trust because doctors’ diagnoses that can affect patients’ health can differ depending on the sensed health information [3]. In particular, in the case of patients in critical condition, their medical devices must provide a high level of trust.

**Emergent access to medical devices.** One important challenge for medical devices is the capability of emergent access to medical devices [12,141]. Basically, strict authentication and authorization are required to protect the security of medical devices; however, medical staff should be able to access patients’ medical devices in an emergency if patients are unavailable or lose consciousness. This functionality is critical by dint of being directly related to the patients’ life.

### 7.3. Medical Network

To provide security and privacy for e-health data with limited resources, security solutions in the surveyed studies have generally been focused on efficiency and simplicity. Detailed research trends for the security and privacy of the medical networks are as follows.

**Efficient and secure transmission.** Most security and privacy studies applied cryptography to provide data confidentiality and integrity, which are the most important requirements when transmitting e-health data via a medical network. Meanwhile, efficiency should also be considered, since medical networks are resource-constrained in terms of computational costs, bandwidth, energy constraints, and so forth. Signcryption, online/offline encryption, compressive sensing, and batch operation are representative techniques to improve efficiency in the medical network research area.

**Privacy-preserving data aggregation with homomorphic encryption.** Once e-health data has been encrypted, only legitimate participants such as the data owner and medical staff must be able to decrypt the data. However, there is a case that needs to decrypt e-health data where the data has been aggregated and processed in the middle of the network. In this case, most studies adopted homomorphic encryption to protect data confidentiality by computing on ciphertext directly. In other words, sensitive e-health data can be processed without data decryption by means of homomorphic encryption. The studies utilized several cryptosystems that have the homomorphic property (e.g., ElGamal, Paillier, and Boneh–Goh–Nissim).

**Certificateless cryptography techniques.** Public-key cryptography is required to provide authenticity and non-repudiation; however, conventional public-key infrastructure has some shortcomings such as the key escrow problem and complex certificate management. Therefore, certificateless public-key cryptography has been utilized in various studies to eliminate both the key escrow problem and certificate management. In the surveyed studies, certificateless cryptography techniques were combined with diverse security solutions such as authentication, signature, and signcryption.

**Mutual authentication.** Mutual authentication is essential to ensure that e-health data are transmitted from the right patient and is received by the desired medical staff. For example, mutual authentication is required to ensure the integrity of a patient’s e-health data and the doctor’s prescription in a remote e-healthcare service. If the patient’s data or doctor’s prescription has been compromised, this can lead to a critical situation. According to our survey, authentication studies in the medical network research area were generally categorized into lightweight authentication schemes that are efficient for resource-constrained environments and anonymous authentication schemes that focus on privacy preservation during the authentication process.

Since medical networks transmit e-health data, strong security solutions should be applied. However, existing strong security solutions cannot be directly adopted because of the limited resources of medical networks. Maintaining a reasonable tradeoff between the strength and efficiency of the security solution, therefore, is the main challenge in the medical network research area. Open challenges for the security and privacy of the medical network areas are as follows.

**Resource constraint.** Conventional security solutions cannot be directly applied to medical networks such as WBANs and IoMTs since they are resource-constrained in terms of computing power, memory capacity, and bandwidth. Although resources are limited, security solutions such as cryptography and authentication are indispensable for network security. Efficient security solutions have been studied; however, it remains difficult to provide sufficient security and reliability for highly sensitive e-health data with limited resources compared to other environments. Making a better tradeoff between security and efficiency is a challenging problem.

**Conditional privacy preservation.** Patient privacy must be preserved. The identities of patients in medical networks are anonymous so that adversaries cannot specify patients’ real identities. However, a user’s real identity should be discernible in some cases so that a trusted provider can trace them. Based on this traceability, an adversary who has malicious purposes can be identified by a trusted server. To this end, Ji et al. [60] and Yang et al. [63] designed a conditional identity preservation scheme; however, some studies argued that identities must be anonymous in any circumstances to provide untraceability. Despite having the advantage that an adversary or malicious user can be traced, a conditional privacy-preserving scheme also has the disadvantage that an insider can abuse its functionality. In the near future, the tradeoff between the advantages and disadvantages should be studied in detail. In addition, an abuse prevention scheme should be studied for the conditional privacy-preserving function.

### 7.4. Edge, Fog, and Cloud

Based on the sufficient resources, security and privacy studies that deploy edge, fog, and cloud computing have focused on useful functionalities such as secure data outsourcing and sharing. Detailed descriptions for the research trends on the edge, fog and cloud computing are as follows.

**Secure outsourcing.** A cloud-based service provider is an honest-but-curious model. That is, although it follows the security protocols and solutions, it could also extract some private information during the process. Therefore, outsourcing e-health data to a cloud-based service provider must be secure and transparent. The data owner must be able to check the integrity of the outsourced data because it could be altered and deleted in the cloud. Efficient and comprehensive data auditing schemes that provide public verifiability such as PDP and PoR will become more important as the use of cloud-based healthcare services increases.

**Secure sharing of e-health data.** E-healthcare systems collect individuals’ e-health data. Nowadays, big e-health data are considered a valuable resource for diverse purposes. The e-health data both provide personalized healthcare services (e.g., remote health condition monitoring, diagnosis, and treatment) and can be used to study diseases. To this end, security and privacy must be required to share highly sensitive e-health data. The data requester (e.g., a patient) and receiver (e.g., a doctor) must be authenticated and authorized and certain cryptography schemes such as proxy re-encryption, attribute-based encryption, and searchable encryption were used to securely share data. In addition, blockchain is a promising security solution to ensure data integrity and accountability, which are important security requirements for e-health data sharing.

**Fine-grained access control using attribute-based encryption.** The cloud is mostly used to store e-health data; therefore, it is inevitable that the cloud will store big e-health data. In state-of-the-art studies, fine-grained access control schemes have been widely proposed to protect e-health data based on ABE. Since ABE encrypts and decrypts data with diverse attributes, it is very flexible in terms of realizing fine-grained access control. Moreover, recent studies have adopted additional security solutions such as blockchain, trust management, dynamic auditing, and online/offline cryptography schemes to support various security requirements.

Various studies have been conducted, however, there are some open challenges that should be solved in the near future. In particular, as e-health service providers increase, a need for secure data sharing and interoperability between the providers have been increasingly grown. The rest of this section presents the open challenges.

**Improvement of usability for secure data sharing.** Recently, secure data sharing studies have been conducted, and a service that shares e-health data has been launched. However, it still lacks usability because of security and privacy concerns. For example, the Healthcare Big Data Platform [4], a South Korean e-health data sharing service, has been launched for the public use of patients’ health data; however, it takes a long time to use the data because of complex request processes that consist of eight phases. In addition, researchers can request just limited data that is registered in the data catalog; therefore, enhancing the usability of sharing e-health data, while ensuring the security and privacy of data, is a challenging problem.

**Secure interoperability among multiple e-health data providers.** As e-healthcare services increase, patients’ e-health data have been widely distributed in various service providers. In a real scenario, data can be distributed despite belonging to the same patient because a patient can use various service providers. Therefore, secure interoperability among multiple data providers is required to provide user-centric data governance for distributed data. In other words, users should be able to search, use, and manage their distributed data across heterogeneous data providers based on secure interoperability. To this end, end-to-end security and mutual authentication must be established among providers and other security solutions including fine-grained access control and access policy translation should be studied with the consideration of newly emerging security concerns and requirements for secure interoperability.

**Complete and conditional anonymity.** E-health data and user identities should be anonymized depending on the situation. To this end, some studies provide complete anonymity in which data cannot be identified in any situation, while other studies provide conditional anonymity where data can be identified in a special situation. There is controversy among researchers over whether anonymity must not be breached in any case or conditional anonymity is required in a few cases to identity attackers. The two types of anonymization studies have different advantages: strong privacy, and conditional traceability, respectively; however, complete anonymity cannot provide traceability while conditional anonymity cannot provide a high level of privacy since identities can be revealed. Therefore, finding anonymization schemes that can provide high privacy while considering traceability is an open challenge.

**In-depth security analysis for edge and fog computing.** Storing and processing e-health data in cloud computing are prominent research trends; however, edge and fog computing paradigms have been emerging because of the latency-sensitive and context-awareness requirements [142,143]. In particular, edge and fog computing can be utilized to develop real-time medical services that support space- and time-awareness and can also preprocess and analyze e-health data in a secure and private manner to reduce the communication cost between medical devices and the cloud. Since the edge and fog computing paradigms have recently been integrated into modern e-healthcare systems, in-depth security analysis that considers real e-healthcare scenarios will be required in the near future to identify new types of security concerns and requirements.

**A lack of open-source-based edge, fog, and cloud computing platforms.** According to our survey, few studies have implemented the proposed security solutions based on real edge, fog, and cloud environments. The implementation of security solutions based on real environments would be valuable work to demonstrate their feasibility and real performance; however, it is difficult to build environments based on edge, fog, and cloud computing. Therefore, building open-source-based platforms for edge, fog, and cloud computing that can simply be used to implement and evaluate the proposed security solutions remains an open challenge.

## 8. Conclusions

Innovations in e-health systems present a double-edged sword. Although they provide advanced healthcare services, there are increasing security concerns with regard to e-health data, which is highly sensitive information. Therefore, we have surveyed recent studies on security and privacy issues related to e-health data according to the target domains, that is, e-health data, medical devices, medical networks, and edge/fog/cloud computing. In this survey, we identified the security concerns and requirements that are commonly mentioned in studies and provided promising security solutions. In particular, based on the literature review, we developed four taxonomies on the security concerns, requirements, and solutions for each component of modern e-health systems. Furthermore, we analyzed the strengths and weaknesses of the surveyed studies, and provided recent research trends and open challenges on security and privacy for the e-health systems. Compared to other surveys, we comprehensively reviewed the security and privacy issues for e-health data including the surrounding environments, that is, medical devices, medical networks, and edge/fog/cloud computing. Finally, as e-health systems become more complex across various layers and data have been exchanged among different domains, secure interoperability among heterogeneous e-health systems should be specifically researched in the near future. 

## Figures and Tables

**Table 1:** Journal sources.

Publisher	Journal	IF	SJR
IEEE	IEEE Access	3.37	0.59
	IEEE Systems Journal	3.93	0.86
	IEEE Journal of Biomedical and Health Informatics	5.77	1.29
	IEEE Transactions on Information Forensics and Security	7.18	1.61
	IEEE Internet of Things Journal	9.47	2.08
	IEEE Transactions on Industrial Informatics	10.21	2.50
	IEEE Transactions on Image Processing	10.86	1.78
	IEEE Transactions on Parallel and Distributed Systems	2.69	0.76
	IEEE Transactions on Services Computing	5.7	0.97
	IEEE Transactions on Cloud Computing	8.22	1.21
	IEEE Reviews in Biomedical Engineering	-	1.64
	IEEE Transactions on Biomedical Engineering	4.54	1.15
	IEEE Transactions on Wireless Communications	7.02	2.01
	IEEE Transactions on Biomedical Circuits and Systems	3.83	1.02
	IEEE Journal on Selected Areas in Communications	9.14	2.99
	IEEE Transactions on Dependable and Secure Computing	7.33	1.27
ACM	Communications of the ACM	4.65	0.97
IEEE/ACM	IEEE/ACM Transactions on Computational Biology and Bioinformatics	3.71	0.75
	IEEE/ACM Transactions on Networking	3.56	1.02
Elsevier	Future Generation Computer Systems	7.19	1.26
	Computer Methods and Programs in Biomedicine	5.43	0.92
	International Journal of Medical Informatics	4.05	1.12
	Computers in Biology and Medicine	4.59	0.88
Springer	Journal of Medical Systems	4.46	0.69
	Neural Computing and Applications	5.61	0.71
	Journal of Big Data	-	1.03
SAGE	Health Informatics Journal	2.68	0.56
JMIR	Journal of Medical Internet Research	2.68	1.45
	JMIR Medical Informatics	2.96	-
	JMIR mHealth and uHealth	4.77	1.36
Oxford University Press	Europace	5.21	2.12
Taylor & Francis	Expert Review of Medical Devices	3.17	0.62
Diabetes Technology Society	Journal of diabetes science and technology	-	1.04

**Table 2:** Comparison of the survey papers in terms of the target domains.

Reference	E-Health Data	Medical Device	Network	Edge	Fog	Cloud
Kruse et al. [5]	✔					
Abouelmehdi et al. [6]	✔					
Mohammed et al. [7]	✔					
Aziz et al. [8]	✔					
Zheng et al. [9]		✔	✔			
Wu et al. [10]		✔				
Yaqoob et al. [3]		✔	✔			
Kintzlinger et al. [11]		✔	✔			
AlTawy et al. [12]		✔	✔			
Yaacoub et al. [13]		✔	✔			
Sun et al. [14]	✔	✔	✔			✔
Chenthara et al. [15]	✔		✔			✔
Yüksel et al. [16]			✔			✔
Wazid et al. [17]	✔	✔	✔			
Razaque et al. [18]	✔	✔	✔			✔
**Our Survey**	✔	✔	✔	✔	✔	✔

**Table 3:** Summary for e-health data security and privacy studies.

Ref.	Target Security Concern	Target Security Requirement	Security Solution	Strength	Weakness
[21]	- Unauthorized access - Data disclosure - Data tampering	- Access restriction - Confidentiality - Anonymity - Accountability	- Access Control - Anonymization	Anonymization and access control according to the sensitivity of e-health data	The current anonymization method has some vulnerabilities that could lead to re-identification
[22]	- Unauthorized access - Data disclosure - Data loss - Data tampering - Data forgery	- Access restriction - Confidentiality - Integrity - Availability - Accountability	- Access Control - Blockchain (Hyperledger Fabric)	No single point of failure (SPoF) problem and the emergency scenario is considered	Emergency access permission could be abused and access control requires a transaction fee
[23]	- Unauthorized access - Data disclosure - Data loss - Data tampering - Data forgery	- Access restriction - Confidentiality - Integrity	- Access Control - Blockchain (Ethereum)	No SPoF problem and employing an off-chain scaling method to solve the scalability problem of blockchain	Lack of fine-grained access control and access control requires a transaction fee
[24]	- Unauthorized access - Data disclosure - Data loss - Data tampering - Data forgery	- Access restriction - Confidentiality - Integrity - Accountability	- Access Control - AES - Blockchain	A user can revoke permission to access their e-health data from medical staff at any time	Symmetric key was shared to control access; therefore, once a key has been shared with someone or compromised, the key should be updated and related e-health data should be re-encrypted
[27]	- Data disclosure	- Confidentiality	- AES - RSA - Steganography	Discrete wavelet transform is compatible with compression and has resistance to geometric distortions	IoT is a target environment; however, AES and RSA that require high computational power were used
[28]	- Data disclosure	- Confidentiality	- Quaternion-based Encryption	Fast computation speed for the encryption of a large volume of e-health data	The computation speed could be increased if the decomposition process were omitted
[29]	- Data disclosure	- Confidentiality	- ECEM-based Encryption	Differential attack resistance	Performance should be evaluated on diverse medical images
[36]	- Data disclosure - Data tampering - Data loss - Data forgery	- Confidentiality - Integrity - Anonymity - Accountability	- AES - Blockchain	No SPoF problem and medical data can be securely preserved with the blockchain	The transaction fee is relatively high compared to conventional data storage
[37]	- Data disclosure - Data tampering - Data loss - Data forgery	- Access restriction - Confidentiality - Integrity - Anonymity - Accountability	- Access Control - Blockchain	Efficient consensus mechanism and access control protocol for e-health data were also proposed	Access should be able to be delegated to related medical staff or other people in a secure manner for flexible data sharing
[38]	- Data disclosure - Data tampering - Data loss - Data forgery	- Integrity - Accountability	- Blockchain	Blockchain only records URLs instead of medical images that have a large data size	Since real images are stored in the hospital’s database, both the blockchain and endpoints should be properly protected
[39]	- Data disclosure	- Confidentiality	- Rijndael encryption - Steganography	Fast processing time and high embedding capacity based on LSB	Message capacity could be increased with noise cancellation and data reduction
[40]	- Data disclosure - Data tampering	- Confidentiality - Integrity	- AES - Steganography	It considered both confidentiality and integrity and had high capacity, robustness, and imperceptibility	An error control mechanism should be adopted for a robust steganography method
[41]	- Data disclosure - Data tampering - Data forgery	- Confidentiality - Integrity	- Watermarking	Resistance to sharpening and blurring attacks while maintaining acceptable imperceptibility	A tracking key that makes the proposed scheme reversible has to be transmitted with each medical image
[42]	- Data disclosure - Data tampering - Data forgery	- Confidentiality - Integrity	- Watermarking	Proposes an effective scheme to localize and restore tampered pixels and regions	Various tampering attacks on image resizing, skewing, and rotating should be studied

**Table 4:** Summary for medical device studies on security and privacy.

Ref.	Target Security Concern	Target Security Requirement	Security Solution	Strength	Weakness
[49]	- Unauthorized access - Data breach - Network attack	- Access restriction - Confidentiality - Integrity - Resistance to network attack	- Mutual authentication	Both local authentication and remote authentication are considered for cloud-assisted wearable devices	Details of implementation and security analysis against diverse possible attacks are insufficient
[51]	- Unauthorized access - Network attack	- Access restriction - Availability - Resistance to network attack	- Biometric-based authentication (fingerprint)	A fingerprint-based authentication model is proposed that would be suitable for IMDs	Fingerprints cannot be re-generated; therefore, fingerprint-based security solutions must consider other solutions to protect fingerprints since fingerprint must then never be leaked
[52]	- Unauthorized access - Network attack	- Access restriction - Availability - Resistance tonetwork attack	- Biometric-based authentication (ECG and fingerprint)	A strong authentication scheme is proposed using two-factors, ECG, and fingerprint	A backdoor can be abused by insiders or attackers
[54]	- Data breach - Network attack	- Confidentiality	- ECG- and OTP-based encryption	OTP, which is theoretically unbreakable, is used and it does not require key distribution, storage, revocation, or refreshment using random ECG signals as OTP keys	Proposed scheme requires an additional device to capture ECG signals and the ECG- and OTP-based encryption scheme may not be suitable for resource-constrained IMDs

**Table 5:** Summary for security and privacy study analysis on medical network.

Ref.	Target Security Concern	Target Security Requirement	Security Solution	Strength	Weakness
[59]	- Collusion attack - MITM attack	- Scalability - Confidentiality - Availability - Integrity - Authenticity	- AES - CP-ABE - Access Control	Efficient approach of using ABE by encrypting a symmetric key, RSK, instead of the whole data	Security for e-health data depends on a secure socket layer (SSL) that could not be fully adopted in a resource-constrained WSN environment
[55]	- Eavesdropping - Tracking - Spoofing attack	- Confidentiality - Availability (reliability)	- AES - ABE - Authentication - Access Control	Flexible privacy protection strategies according to three trust levels of a user or node	Security could easily be threatened if a node that has a high trust level is compromised
[57]	- Eavesdropping - Impersonation attack - Replay attack - DoS attack	- Confidentiality - Integrity - Authenticity - Non-repudiation - Lightweight - Forward security	- ECC - Mutual Authentication	Efficient authentication protocol using a non-pairing operation and ECC-based scheme	The required computation cost is still high for resource constrained WBAN nodes because of the certificateless scheme
[60]	- Replay attack - Impersonation attack - MITM attack	- Anonymity - Unlinkability - Forward Secrecy	- ECC - Mutual Authentication	Conditionally anonymous authentication to trace a malicious user and batch authentication for efficiency	Conditional traceability could be abused by an insider; however, there is no mention of this drawback
[68]	- Replay attack - Impersonation attack - Spoofing attack - DoS attack - Location tracking - MITM attack	- Confidentiality - Anonymity - Availability - Forward secrecy - Scalability	- ECC - Mutual Authentication	Low computation cost and communication overhead	The communication between tag and reader was insecure
[69]	- Eavesdropping - Replay attack	- Confidentiality - Authenticity - Lightweight	- ECC - CLSC - Authentication	Efficient scheme based on ECC and signcryption	Anonymity should be considered to ensure patient privacy
[71]	- Eavesdropping - Replay attack	- Confidentiality - Lightweight	- ABE	Efficient ABE based on online/offline encryption techniques and ABF for access control policy to protect the privacy of users’ attributes	ABF could hinder the encryption performance
[63]	- Eavesdropping - Replay attack - Impersonation attack - Tracking attack	- Confidentiality - Anonymity - Authenticity	- ABE - Access Control	Low computation cost for EHR encryption/decryption	Pairing operation that cause high computation cost is required
[73]	- Eavesdropping - Replay attack	- Confidentiality - Integrity - Authenticity	- Pairing-based HE - Aggregate Signature	Data confidentiality is preserved while data aggregation and batch verification are performed for efficiency	Requires exponentiation and pairing operations that cause a high computation cost
[74]	- Eavesdropping - Replay attack - Impersonation attack	- Confidentiality - Forward secrecy - Backward secrecy	- HE - Key Distribution	Direct communication between a patient’s mobile device and medical devices is possible	Diagnosis reliability should be provided
[64]	- Eavesdropping - Replay attack - Collusion attack	- Availability (Fault Tolerance) - Collusion resistance	- BGN cryptosystem	Differential attack and privacy are considered	The BGN cryptosystem has a small plaintext space for e-health data
[80]	- Eavesdropping - Replay attack	- Anonymity - Confidentiality - Integrity - Non-repudiation	- CLSC - Authentication - Access Control	Key escrow resilience and elimination of certificate management based on certificateless access control	Requires exponentiation and pairing operations that cause high computation cost
[82]	- Eavesdropping - Impersonation attack	- Confidentiality - Integrity - Anonymity - Lightweight - Unlinkability - Forward secrecy	- CLGSC	Key escrow resilience and low computation cost by eliminating pairing operations	Requires a relay selection strategy to improve transmission efficiency and reliability
[75]	- Eavesdropping	- Confidentiality - Integrity	- HE	Eavesdropping in wireless environments could be mitigated by dividing data	A sensor node in WSNs could not use HE because of the resource constraint
[62]	- Eavesdropping - Replay attack - Impersonation attack - MITM attack	- Integrity - Non-repudiation - Forward secrecy	- Mutual Authentication - Key agreement based on Chebyshev chaotic map	The major advantage is that it provides continuous remote patient supervision that can improve patient health	SPoF can be posed because of the centric medical cloud that manages all patients’ health data
[83]	- Eavesdropping - Replay attack - Impersonation attack - MITM attack	- Anonymity - Authenticity - Forward secrecy	- Mutual Authentication	Medical data cannot be tampered with and is untraceable by means of a blockchain	Using the cloud as a central database of medical data can cause SPoF. This drawback could weaken the advantages of blockchain
[84]	- Replay attack - Eavesdropping - Impersonation attack - MITM attack	- Confidentiality - Integrity - Availability - Anonymity - Lightweight - Unlikability - Forward secrecy - Backward secrecy	- Mutual Authentication	Very low computation cost and energy consumption	Mutual authentication is only considered between the WBAN and controller nodes
[85]	- Eavesdropping - DoS attack - Impersonation attack - Tracking attack	- Confidentiality - Anonymity - Authenticity - Unlinkability	- Authentication - Access Control	A lightweight authentication protocol for resource-constrained RFID tags	When an RFID reader gets an authentication response from a tag, all group keys should be used to decrypt the response until it succeeds
[68]	- Replay attack - DoS attack - Impersonation attack - MITM attack - Spoofing attack - Tracking attack	- Confidentiality - Availability - Anonymity - Forward secrecy - Scalability	- Mutual Authentication	Low computation cost and communication overhead and solves some security flaws of previous authentication schemes	The session key should be generated for security between a tag and reader because the secure channel between the tag and reader was not established
[65]	- Eavesdropping - Replay attack - DoS attack - Tracking attack	- Confidentiality - Anonymity - Forward security	- Mutual Authentication	Low computation cost	Communication cost is a little high compared to other studies
[66]	- Eavesdropping - Replay attack - Impersonation attack - Tracking attack	- Confidentiality - Integrity - Forward secrecy - Backward secrecy	- Mutual Authentication	Low computation power requirement for RFID tag based on the proposed lightweight MRot(x,y) function	It could be vulnerable to secret disclosure attack
[87]	- Replay attack - DoS attack - Spoofing attack	- Confidentiality - Integrity - Availability - Authenticity - Non-repudiation	- Flexible Access Control	The proposed access control model supports a flexible access control policy based on the BTG concept	ID and password are required when the BTG policy is applied, and the proposed access control model did not provide anti-tampering measures
[58]	- Eavesdropping	- Confidentiality	- Compressive sensing	Chaotic CS more energy-efficient and secure than traditional CS	Encrypted data might be easily decrypted if an adversary takes a measurement matrix because the encryption is performed with the same matrix
[56]	- DoS attack	- Availability	- Traceback technique	Lightweight to be applied in a WBAN environment	The proposed technique is only based on WBAN and MAC header and the number of bytes in the DPPM label depends on the network topologies

**Table 6:** Summary for the security and privacy studies on edge, fog, and cloud computing.

Ref.	Target Security Concern	Target Security Requirement	Security Solution	Strength	Weakness
[92]	- Data disclosure	- Confidentiality - Authenticity	- Identity-based encryption - Proxy re-encryption	Efficient identity-based cryptographic schemes were proposed and adopted in a medical domain to provide implicit authentication of the public key and simple certificate management	There is a key escrow problem because it uses a centralized server
[93]	- Unauthorized access - Data disclosure - Malicious insider	- Access restriction - Confidentiality	- File hierarchy CP-ABE	Considered file hierarchy in the CP-ABE for efficient encryption in terms of storage and time cost	Pairing operations consume high computing power
[94]	- Unauthorized access - Data disclosure	- Access restriction - Confidentiality	- IBE - CP-ABE	A patient-controlled CP-ABE scheme was proposed considering emergency situations	Identity authority has the capability to generate an emergency key to decrypt a patient’s data, which could be compromised by an attacker or abused by an insider.
[95]	- Unauthorized access - Data disclosure	- Access restriction - Confidentiality - Anonymity - Authenticity	- CP-ABE - Signcryption - Access control	Promising cryptographic technology, CP-ABSC, was proposed for fine-grained access control allowing the secure sharing of e-health data in cloud computing	Revocation scheme of a user and attributes should be considered and Rao claimed that it cannot provide confidentiality [76]
[96]	- Unauthorized access - Data disclosure	- Access restriction - Confidentiality - Anonymity - Authenticity - Integrity and public verifiability	- CP-ABE - Signcryption - Access control	The proposed CP-ABSC also supports signcryptor privacy and public verifiability, which are important security requirements in cloud environments	A high computation cost is required for designcryption because of the pairing operations
[99]	- Data breach - DoS attack - Single point of failure	- Confidentiality - Availability - Anonymity - Flexibility - Scalability	- Homomorphic encryption	A hybrid secure sharing scheme for e-health data is considered to cover both the advantages of centralized and decentralized approaches	Collusion attacks between cloud providers and users should be considered
[103]	- Unauthorized access - Data breach - Eavesdropping	- Access restriction - Confidentiality	- Searchable encryption - Proxy re-encryption	The proposed SE scheme allows a patient to delegate access permissions to others to search and decrypt the patient’s data, which is automatically revoked after a time limit	Access permissions are revoked after the time limit expires; however, the delegatee can use the data since they have already obtained it in plaintext by decrypting the data within the time limit
[104]	- Unauthorized access - Data breach - Eavesdropping	- Access restriction - Confidentiality - Integrity	- Searchable encryption - Homomorphic encryption	The proposed scheme can perform privacy-preserving data sharing with key range search and multiple keyword search in e-health systems	A post management scheme for e-health data may be required after searching and using the data
[105]	- Unauthorized access - Data breach - Single point of failure	- Access restriction - Confidentiality - Integrity - Authenticity - Accountability	- Searchable encryption - Blockchain	A searchable encryption scheme was used with the blockchain to provide integrity, anti-tampering, and accountability for e-health data sharing	Since the data are stored in the public cloud, it may require additional security solutions for the data
[106]	- Unauthorized access - Data breach	- Access restriction - Confidentiality	- Searchable encryption - Order-preserving symmetric encryption	An efficient query over multiple data providers’ data	The cloud is required since the proposed scheme may not be suitable for resource-limited devices (e.g., smartphones)
[107]	- Unauthorized access - Data breach - Eavesdropping	- Access restriction - Anonymity - Authenticity - Resistance to network attacks	- Mutual authentication	A mutual authentication scheme for telecare systems providing patient anonymity	The time required for mutual authentication is slower than the base scheme [114]
[108]	- Unauthorized access - Data breach - Malicious insider - Eavesdropping	- Access restriction - Anonymity - Authenticity - Accountability - Resistance to network attacks	- Mutual authentication - Secret sharing	Proposes privacy-preserving mutual authentication for mobile edge-cloud architecture	The communication cost should be reduced for resource-constrained medical networks such as IoMTs and WBANs
[109]	- Unauthorized access - Data breach - Malicious insider - Eavesdropping	- Access restriction - Anonymity - Authenticity - Resistance to network attacks	- Mutual authentication - Blockchain	The proposed scheme is suitable for big e-health data because of its cost-efficiency	More practical security threats to the proposed scheme are identified and analyzed
[110]	- Unauthorized access - Data breach - Single point of failure - Network attacks	- Access restriction - Confidentiality - Integrity - Resistance to network attacks	- Mutual authentication - Access control	Edge computing is utilized to securely provide local services to users in a certain area (e.g., a hospital)	More security concerns including location tracking attack, which emerge when adopting hybrid computing, should be discussed
[111]	- Unauthorized access - Data breach - Eavesdropping	- Access restriction - Anonymity - Authenticity - Resistance to network attacks	- Anonymous authentication	Complete privacy and anonymity are provided to users from adversaries and an authentication server	Traceability may be conditionally provided when security incidents happen to track attackers
[112]	- Unauthorized access - Data breach - Malicious insider - Eavesdropping	- Access restriction - Anonymity - Authenticity - Resistance to network attacks	- Traceable authentication	The proposed scheme provides conditional identity traceability and is efficient in terms of communication cost and energy for resource-constrained devices	Providing conditional traceability might be abused; therefore, a prevention method for such abuse is required
[114]	- Unauthorized access - Data breach	- Access restriction - Confidentiality - Availability - Flexibility	- Proxy re-encryption - Access control - Trust and reputation	The proposed scheme can provide flexible access control based on trust and reputation, even when the data owner is unavailable or cannot make access decisions	The level of trust and reputation might be ambiguous factors to decide access to highly sensitive e-health data
[115]	- Unauthorized access - Data breach - Single point of failure	- Access restriction - Confidentiality - Integrity - Availability	- Proxy re-encryption - Access control - Dynamic data auditing	It is suitable for resource-constrained devices and solves the cloud reciprocity problem	User anonymity should be considered
[116]	- Unauthorized access - Data breach - Malicious insider - Network attacks	- Access restriction - Confidentiality - Anonymity - Authenticity - Resistance to network attacks	- Authentication - Access control	It is designed to be lightweight for resource-constrained devices and the access control scheme	Conditional traceability may be required and the cloud provider can abuse the ability to manage the group key
[117]	- Unauthorized access - Data breach	- Access restriction - Confidentiality	- Online/offline CP-ABE - Access control	An efficient online/offline CP-ABE scheme is proposed for resource-constrained devices in mobile cloud computing	Bilinear pairings used in the proposed scheme pose a high cost that hinders efficiency
[118]	- Unauthorized access - Data breach	- Access restriction - Confidentiality	- ABE - Access control	An efficient ABE scheme is proposed to reduce the time required to encrypt and decrypt data that has the same access policy	Privacy for access policies should be supported
[119]	- Unauthorized access - Data breach	- Access restriction - Confidentiality - Flexibility - Scalability	- Access control	A flexible access control model is proposed using situation-based access policy	More fine-grained access control could be considered with an attribute-based access control model
[120]	- Unauthorized access - Data breach	- Access restriction - Confidentiality - Anonymity - Integrity	- CP-ABE - Access control - LSSS	Privacy for access policy is ensured by hiding the entire access policy with attributes	Resource-constrained medical devices are not suitable for the proposed scheme since hiding the access policy requires greater costs
[121]	- Unauthorized access - Data breach	- Access restriction - Confidentiality - Flexibility	- Proxy re-encryption - ABE - Access control	A method to transform a private cloud’s access policy to the access policy of a public cloud	The proposed scheme might be implemented to show feasibility, performance, etc.
[122]	- Unauthorized access - Data breach	- Access restriction - Confidentiality	- CP-ABE - LSSS	A method for updating an access policy in ciphertext that reduces the computational cost	The proposed scheme incurs additional overhead to cloud
[100]	- Data breach - Single point of failure	- Confidentiality - Scalability	- Fully homomorphic encryption	E-health data can be securely aggregated and monitored in real-time using edge computing	A performance evaluation for the fully homomorphic encryption would be helpful and show the feasibility of the proposed framework
[123]	- Unauthorized access - Data breach	- Access restriction - Confidentiality	- Access control	Fog computing is adopted to reduce the communication cost of IoMTs	The access control may not be sufficiently fine-grained for diverse e-healthcare systems
[124]	- Unauthorized access - Data breach	- Access restriction - Confidentiality - Integrity	- ABE - Access control	A consensus-based access control scheme is proposed	If the number of nodes that participate in the consensus is insufficient, an attacker could control access to e-health data
[127]	- Data breach	- Integrity and public verifiability	- Identity-based auditing	It provides comprehensive auditing in terms of data origin, type, and consistency	Dynamic data auditing is unsupported
[128]	- Data breach - Malicious insider	- Confidentiality - Integrity and public verifiability - Authenticity	- Identity-based auditing	A privacy-preserving identity-based auditing scheme is proposed	A manager who may have malicious purposes can check whether private information exists in the e-health data
[129]	- Data breach	- Confidentiality - Integrity and public verifiability - Anonymity	- Certificateless PDP	The proposed scheme is efficient and can prevent the key escrow problem	The true identity of a malicious user or attacker may be identified and traced for accountability
[130]	- Unauthorized access - Data breach - Single point of failure	- Access restriction - Confidentiality - Integrity - Availability - Accountability - Flexibility	- Access control - Blockchain	The proposed framework was implemented to show feasibility based on Ethereum	An EHR manager manages the keys to decrypt e-health data; therefore, it can be misused or targeted by an attacker
[131]	- Unauthorized access - Data breach - Single point of failure	- Access restriction - Confidentiality - Integrity - Availability - Accountability	- Searchable encryption - Proxy re-encryption - Authentication - Access control - Blockchain	An efficient and reliable consensus mechanism—proof of authorization—was proposed for this system	The proposed scheme cannot ensure full ownership of outsourced e-health data since data providers exist
[132]	- Unauthorized access - Data breach - Single point of failure	- Access restriction - Confidentiality - Integrity - Authenticity - Accountability	- ECC - Access control - Blockchain	A user-centric e-health data management system is proposed	Permission delegation for e-health data may be supported to share e-health data and a user must manage the key themself
[133]	- Unauthorized access - Data breach - Single point of failure	- Access restriction - Confidentiality - Integrity - Availability - Anonymity - Scalability	- Blockchain - Tor	A secure therapy application utilizing MEC is proposed and implemented	The study did not provide in-depth security analysis for the proposed MEC-based therapy application
[136]	- Unauthorized access - Data breach	- Access restriction - Confidentiality - Authenticity	- ECC - Mutual authentication - Decoy	A security model is proposed that utilizes a decoy technique in e-healthcare cloud using fog computing	Diverse network attacks (e.g., MIMT) should be discussed to prove that the proposed key agreement protocol is highly secure
